# Investigating the role of structural wall stress in aortic growth prognosis in acute uncomplicated type B aortic dissection

**DOI:** 10.1007/s10237-025-02031-9

**Published:** 2025-12-28

**Authors:** Minliang Liu, Yuhang Du, Hannah L. Cebull, Yuxuan Wu, Adam Mazlout, Asanish Kalyanasundaram, Rishika Agarwal, Hai Dong, Marina Piccinelli, John N. Oshinski, John A. Elefteriades, Rudolph L. Gleason, Bradley G. Leshnower

**Affiliations:** 1 Department of Mechanical Engineering, Texas Tech University, 805 Boston Ave, Lubbock, TX 79409 USA; 2 The Wallace H. Coulter Department of Biomedical Engineering, Georgia Institute of Technology and Emory University, Atlanta, GA USA; 3 The George W. Woodruff School of Mechanical Engineering, Georgia Institute of Technology, Atlanta, GA USA; 4 Department of Radiology & Imaging Science, Emory University, Atlanta, GA USA; 5 Aortic Institute at Yale-New Haven Hospital, Yale University School of Medicine, New Haven, CT USA; 6 Division of Cardiothoracic Surgery, Department of Surgery, Emory University School of Medicine, Atlanta, GA USA

**Keywords:** Type B aortic dissection, Aortic growth, Optimal medical therapy, Static determinacy, Wall stress

## Abstract

False lumen expansion is a major factor that determines long-term survival of uncomplicated type B aortic dissection (TBAD). The objective of this study was to investigate whether structural wall stress distributions computed from patient-specific acute TBAD geometries can be used to predict aortic growth rates. Three-dimensional (3D) computed tomography angiography (CTA) of 9 patients with acute uncomplicated TBAD was obtained at initial hospital admission and at their most recent follow-up visits. Patient-specific structural wall stress distributions were computed from the initial baseline CTA using a forward penalty method. Spatially varying blood pressure distributions, derived from computational fluid dynamics (CFD) simulations informed by patient-specific brachial blood pressure (BP) measurements, were incorporated into the forward penalty stress analysis. For 5 patients, transthoracic echocardiography (TTE) data were also available and used to prescribe patient-specific inlet flow conditions in the CFD simulations. Aortic growth rates were quantified and visualized within the 3D TBAD geometries using the initial baseline and follow-up scans. Linear mixed-effects regression analyses were performed to evaluate the spatial correlations between biomechanical markers (structural wall stress, wall shear stress, and pressure) and aortic growth rates. Utilizing initial baseline patient-specific CTA and BP data, along with TTE data when available, the forward penalty analyses revealed hemodynamic and structural mechanics insights of acute uncomplicated TBADs. The linear mixed-effects model indicated that the fixed-effect association between acute structural wall stress and estimated aortic growth rate distributions was statistically significant (*p* = 0.036), which demonstrated that aortic segments experiencing higher structural stress in the acute phase exhibited more rapid growth. Fixed-effect associations were not significant when predicting growth rate using wall shear stress (*p* = 0.88) or pressure (*p* = 0.65) distributions computed from the acute TBAD geometry. Significant Pearson correlation coefficients (*p* < 0.05) were observed between acute structural wall stress and aortic growth rate in all patients. Higher structural wall stress in the acute TBAD geometry was associated with regions of increased aortic growth rates. When modeled as a solid, false lumen thrombus was linked to lower structural wall stress and may have a protective effect against rapid aortic growth. Further studies are needed to investigate the biphasic nature of thrombus. Structural stress, which in this study was derived using the forward penalty approach, may be a novel predictor of aortic growth rate in acute TBAD.

## Introduction

Type B Aortic Dissection (TBAD) is a lethal disease with an incidence of 4 per 100,000 patients per year (Criado 2011). TBAD occurs when a tear develops in the inner lining (intimal layer) of the aorta, causing the layers of the aortic wall to separate (dissect), creating ‘true’ and ‘false’ lumens. Acute TBAD patients are classified as ‘complicated’ based upon the presence of organ malperfusion or aortic rupture; otherwise, they are considered ‘uncomplicated’ (Lombardi et al. 2020). Acute complicated TBAD carries a high mortality rate and is optimally treated with emergent thoracic endovascular aortic repair (TEVAR) (Lou et al. 2019a). Acute uncomplicated TBAD has been traditionally treated with optimal medical therapy (OMT) consisting of aggressive anti-hypertensive and anti-inotropic medications and surveillance imaging. OMT provides excellent short-term survival (83–100%), but is less effective as the dissection ages, with long-term survival rates of 48–66% and overall intervention-free survival rates of < 50% (Afifi et al. 2015; Durham et al. 2015; Leshnower et al. 2017; Nienaber et al. 2013). Recent retrospective reports have demonstrated improved survival with TEVAR compared to OMT in acute uncomplicated TBAD, although no Level I evidence currently exists. The ability to accurately predict aortic growth would allow the identification of patients at high risk for OMT failure that may benefit from early TEVAR and improve long-term TBAD survival.

Previous work has identified demographic and anatomic risk factors associated with OMT failure, aortic growth, and reduced survival in TBAD. These factors include diabetes mellitus, end-stage renal disease, maximum descending aortic diameter ≥ 45 mm, primary intimal tear > 10 mm, the proximity of the primary intimal tear to the left subclavian artery, a patent or partially thrombosed false lumen, and a false lumen diameter > 22 mm (Codner et al. 2019; Evangelista et al. 2012; Lou et al. 2019a; Marui et al. 1999; Onitsuka et al. 2004; Ray et al. 2016 Schwartz et al. 2018; Song et al. 2007; Tsai et al. 2007; Ueki et al. 2014; Van Bogerijen et al. 2014). Although aortic diameter has been the only reproducible risk factor that has emerged from these studies, diameter alone has been shown to be inadequate in predicting the risk of aortic growth, dissection or rupture in patients with aortic disease (Fillinger et al. 2003; Liu et al. 2024; Perez et al. 2023; Vorp et al. 1998). Over the past decade, our group and others have investigated alternative factors that may play an integral role in determining aortic growth in TBAD, namely false lumen hemodynamics and the biomechanical properties of the false lumen wall (Cebull et al. 2024; Dong et al. 2022). Rolf-Pissarczyk et al. provided a comprehensive review summarizing recent advancements in the mechanistic understanding of aortic dissection across pathological, experimental, and in silico modeling domains (Rolf-Pissarczyk et al. 2025). In particular, computational fluid dynamics (CFD) and four-dimensional (4D) flow magnetic resonance imaging (MRI) have been used to assess hemodynamics in TBADs and evaluate patients’ risks (Beaufort et al. 2019; Cebull et al. 2024; Evangelista et al. 2022; Liu et al. 2018a; Marlevi et al. 2021; Polanczyk et al. 2020; Teixido Tura et al. 2022; Zhu et al. 2023). However, these simulation and imaging techniques primarily provide data related to blood flow (e.g., blood pressure, velocity, wall shear stress (WSS)) within the aorta. They do not offer insights into the structural biomechanics of the aortic wall, where the structural stress is several orders of magnitude higher than WSS.

To investigate the role of structural wall stress, conventional fluid–structure interaction (FSI) analysis (Dowell and Hall 2001; Hou et al. 2012) can be employed which provides data on both hemodynamics and structural biomechanics. However, conventional two-way FSI methods require labor-intensive, time-consuming setup and are associated with high computational costs. Completing a single patient’s FSI analysis can take days (Alimohammadi et al. 2015; Munshi et al. 2020). Even with high-performance computing clusters, the process typically takes 18 ~ 45 h (Bäumler et al. 2020), making it impractical for clinical prognostic applications that require rapid feedback to clinicians. Moreover, while two-way FSI with patient-specific tissue material properties is theoretically feasible, determining these patient-specific properties also requires an expensive iterative computation process (Liu et al. 2017, 2018b, 2019a), which greatly reduces the practical value of this complex strategy. In addition, applying the material parameters from one patient toward another can introduce significant uncertainties in the simulations.

Fortunately, our group and others have recently shown that the aortic wall structural stress can be correctly computed by using the principle of static determinacy without knowing patient-specific material properties. This has led to the development of a simple, computationally efficient method known as the forward penalty approach (Liu et al. 2019b; Lu and Luo 2016; Miller and Lu 2013), achieved by enforcing an artificially stiff treatment to the aortic wall. Moreover, recent advancements in nonrigid geometry registration (Bône et al. 2018; Durrleman et al. 2014; Klein et al. 2009) have enabled the quantification of aortic growth from CTA images, described in the literature as vascular deformation mapping (Burris et al. 2022, 2017) or, more generally, registration-based aortic growth mapping (Dux-Santoy et al. 2022; Merton et al. 2024a, b; Teixido-Tura et al. 2024). In this study, we employed the forward penalty approach to compute structural wall stress distributions in patients with acute uncomplicated TBAD. By utilizing static determinacy, the need for two-way solver communication is eliminated, which results in a structural stress computation workflow with significantly reduced computational time. Patient-specific, spatially varying blood pressure distributions, derived from CFD simulations based on inlet flow conditions obtained via clinical transthoracic echocardiograms, were used to compute structural wall stress distributions. Additionally, aortic growth rates were quantified and visualized as 3D heatmaps from CT scans acquired at initial diagnosis and follow-up, using a nonrigid registration technique implemented in Deformetrica (Bône et al. 2018; Durrleman et al. 2014). The purpose of this investigation was to determine whether the spatial distribution of structural wall stress correlated with the spatially varying aortic growth rate in patients with acute uncomplicated TBAD. Developing a tool that could reliably predict aortic growth in acute uncomplicated TBAD could impact the treatment (e.g. OMT vs TEVAR) in these patients and improve survival.

## Methods

### Image segmentation and meshing

**Fig. 1:**
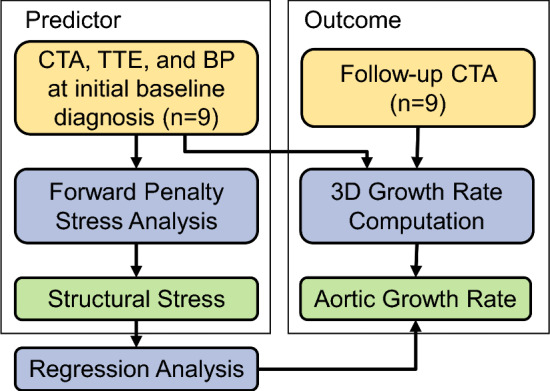
Workflow of study design. CTA: computed tomography angiography; TTE: transthoracic echocardiogram; BP: blood pressure measurement. Forward penalty analysis was performed to compute structural wall stress distribution. Aortic growth rate was quantified on three-dimensional (3D) TBAD geometries. The correlation between structural stress and growth rate was quantified by using regression analysis

**Fig. 2:**
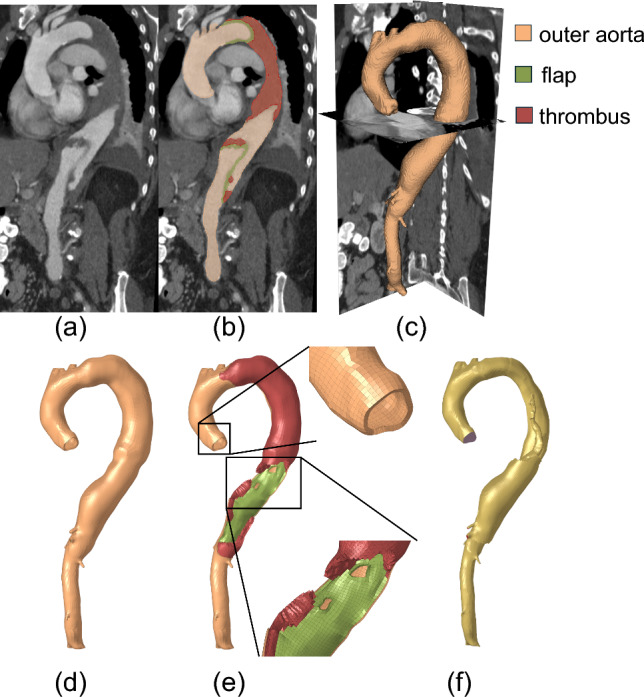
CT image segmentation and mesh generation for a representative patient-specific TBAD geometry. **a** Baseline CT image; **b** segmentation of the entire outer aorta, dissection flap, and thrombosis; **c** 3D surface enclosing the entire outer aorta; **d** generated solid domain mesh of the outer aortic wall, which includes both the true and false lumen walls; **e** cut view of the solid domain mesh with the true lumen wall removed to reveal the dissection flap, intimal tears, and thrombus; and **f** blood-occupied fluid domain mesh

Figure 1 shows the workflow of the overall study design. With approval from the institutional review board (IRB), we retrospectively collected clinical computed tomography angiography (CTA) images, transthoracic echocardiographic data, blood pressure, and demographics of 9 patients who presented with acute uncomplicated TBAD and were initially treated with OMT. Patients with connective tissue disorders were excluded from this study. These data were acquired at the initial admission following the diagnosis of aortic dissection. Additionally, to quantify the rate of aortic diameter expansion, the most recent follow-up CT scans were collected for each patient. The CTA, echocardiogram, and blood pressure were collected as part of standard routine clinical care. ECG gating was not available for the CTAs, and therefore the specific cardiac phase of the CTA acquisition could not be determined. Consequently, motion artifacts were present in some cases, potentially resulting in image blurring. These artifacts can complicate the segmentation of the aortic geometry at a specific cardiac phase, such as diastole, from non-ECG-gated images. To address this, regions affected by motion-induced blurring were considered part of the aorta, which may result in a geometry that approximates the maximum aortic dimensions during the cardiac cycle. To minimize the impact of these artifacts, the CTA was assumed to correspond to the systolic phase for subsequent analysis. Patient-specific 3D geometries of the dissected thoracoabdominal aorta, including brachiocephalic artery, left common carotid artery, left subclavian artery, celiac artery, superior mesenteric artery, the dissection flap, and intraluminal thrombosis, were semi-automatically segmented from the baseline CTA scans of initial hospital admission by the ‘grow from seed’ tool available in the 3D Slicer software. The entire outer aorta encompassing both the true and false lumens, dissection flap, and thrombosis (if present) were segmented separately. In CTA images, Hounsfield unit (HU) values of biphasic thrombosis vary widely (Canellas et al. 2016; Chen et al. 2023), with fluid-phase thrombus typically exhibiting higher HU than solid-phase thrombus. In this study, solid-phase thrombus segmentation was performed using the minimum and maximum HU values of the aortic wall as thresholds. Since all CTAs were contrast-enhanced and the blood pool has higher HU values, early stage fluid-phase thrombus, with HU closer to that of the blood pool, may be excluded by this method. This threshold was applied consistently across all patients with thrombus. Figure 2a–c illustrates the 3D segmented geometries of a representative patient. The segmented dissection flap incorporates tears/fenestrations, which allowed accurate geometric representations for the subsequent simulations. Watertight surface meshes were extracted from the outer aorta, flap and thrombosis segmentations by the ‘model maker’ tool in 3D Slicer.

**Fig. 3:**
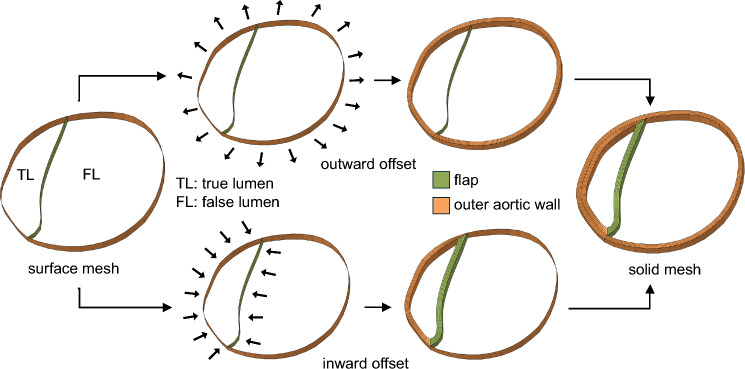
Surface mesh offset workflow used to generate the solid TBAD mesh. A representative cross section of the aorta is shown. An inward offset of 1 mm was applied to the inner surfaces enclosing the true lumen, followed by an outward offset of 1 mm for the entire outer aortic wall. Consequently, the solid mesh has a true lumen wall thickness of 2 mm, with the false lumen wall and dissection flap each having a thickness of 1 mm. These thickness values are based on our group’s experimental work with TBAD tissue specimens (Dong et al. 2022). This approach ensures that the combined thickness of the dissection flap and false lumen wall matches that of the intact aortic wall, representing the wall structure in acute aortic dissections where the freshly dissected flap has just separated from the false lumen wall

**Fig. 4:**
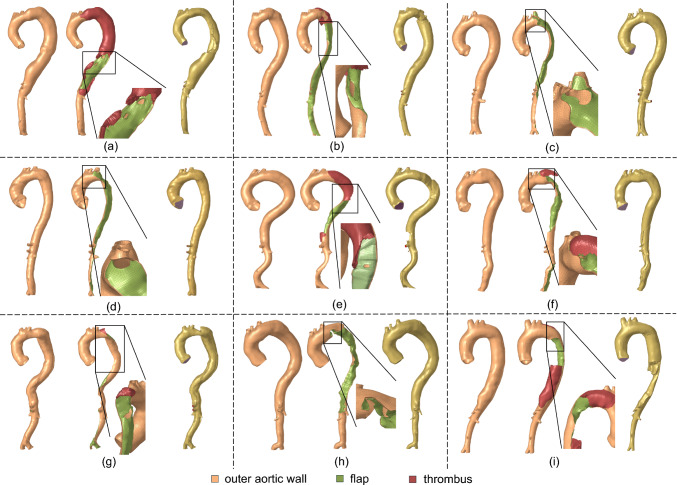
Computational meshes of the solid and fluid domains of all 9 patients involved in this study. Zoomed views of the primary intimal tear are shown. **a** P1; **b** P2; **c** P3; **d** P4; **e** P5; **f** P6; **g** P7; **h** P8; **i** P9. Thrombosis is present in P1, P2, P5, P6, P7, and P9. The orange color was used to depict the aortic wall mesh, which includes both the true and false lumen walls

Following the segmentation process, the surface meshes representing outer aorta, flap and thrombosis segmentations were utilized to generate computational meshes in Altair Hypermesh for fluid and solid computations (see Fig. 2d–f). This involved the following steps: (1) The CT-derived surface mesh of the outer aorta was first used to generate a parametric surface representation. Points at the junctions between the outer aorta and dissection flap surface meshes were manually selected, along with additional sampled points from the flap surface mesh, to construct the flap’s parametric surface. Hence, the edges of the flap surface lie on the outer aorta surface. Due to these shared edges between the aorta and flap surfaces, the resulting surface representation is non-manifold. Based on these parametric surfaces, the outer aorta and dissection flap were remeshed primarily using 2D quadrilateral elements, producing conforming surface meshes, i.e., with shared nodes along the junction between the outer aorta and flap. To determine an appropriate 2D element size, a mesh independence analysis was performed by varying the mesh size from 3 mm to 2.5 mm, 2 mm, and 1.5 mm. The results showed that refining the mesh from 2 mm to 1.5 mm resulted in only a minimal change in the maximum von Mises structural stress (mean absolute percentage error, MAPE: 1.52%). Therefore, an element size of 2 mm was selected for the surface meshes. (2) Solid meshes for the aortic wall and dissection flap were generated by offsetting the 2D surface meshes in surface normal directions. Due to the limited resolution of the CT scans, wall thickness may not be accurately measured. As shown in Fig. 3, an inward offset of 1 mm was applied to the inner surfaces of the aortic wall and dissection flap enclosing the true lumen, followed by an outward offset of 1 mm applied to the entire outer aortic wall. Consequently, the solid mesh has a true lumen wall thickness of 2 mm, with the false lumen wall and dissection flap each having a thickness of 1 mm. These thickness values are based on our group’s experimental work with TBAD tissue specimens (Dong et al. 2022). This approach ensures that the combined thickness of the dissection flap and false lumen wall matches that of the intact aortic wall, which naturally simulates the wall structure in acute aortic dissections where the freshly dissected flap has just separated from the false lumen wall. To ensure sufficient fidelity in capturing transmural variations, the true and false lumen walls and dissection flap were primarily meshed using 4 layers of 3D hexahedral elements (C3D8H). (3) Thrombosis, when present, was meshed using 3D tetrahedral elements. To ensure proper nodal connectivity at the thrombus–false lumen interface, nodes from the inner false lumen surface were duplicated wherever the two regions met. Triangular surface elements were generated from these duplicated nodes and combined with additional surfaces interfacing the blood pool to create a watertight boundary enclosing the thrombus. The thrombus volume was then filled with 3D tetrahedral elements, resulting in a conforming mesh with shared nodes at the thrombus–false lumen interface. Nodal continuity was verified using the ‘Equivalence’ tool in Hypermesh, and any disconnections were manually corrected. This led to approximately 146,707 elements in the solid domain mesh for each patient. (4) Subsequently, the fluid domain occupied by blood was meshed, which is comprised of 8 boundary layers (3D hexahedral elements) and a core (3D tetrahedral elements). To determine the CFD element size, we conducted a mesh independence analysis and analyzed the CFD-predicted peak velocity in a dissection flap fenestration of a representative geometry using various maximum element sizes (4, 3, 2, 1 mm). The mesh at smaller branches and fenestrations is refined by using a factor of 0.25 relative to the maximum element size. The mesh independence test indicated that a CFD mesh consisting of approximately 388,911 elements, with a maximum element size of 2 mm is sufficient. The fluid and solid domain meshes conformed at their interface, allowing for data exchange between the solid and fluid domains. Figure 4 illustrates the solid and fluid domain meshes for the 9 patients included in this study. On average, the segmentation took about 6 h and meshing took about 12 h for a human expert.

### Structural wall stress computation using the forward penalty approach

We employed a reduced-order forward penalty approach to compute structural wall stress distributions on the aortic wall using the baseline CT-derived TBAD geometries. The validity of this forward penalty approach is warranted by the fact that the aortic wall is approximately statically determinate (Liu et al. 2019b), which allows structural stress to be readily computed from image-derived geometries without the need for identifying patient-specific material properties or modeling realistic tissue deformations.

To briefly review the forward penalty approach, we consider a forward elasticity problem in an ex vivo experimental setting, where a surgically resected aortic sample is pressurized in a bulge inflation test. Since the deformation starts from the known undeformed geometry, the resulting deformed configuration and structural stress field depend on the constitutive behavior of the aortic wall. Conversely, in an inverse elasticity problem, where only the deformed geometry is known (e.g., from in vivo clinical CT scans), both the undeformed geometry and the material properties are unknown. If one is known, the other can be inferred. However, in routine CT imaging, which typically captures the aorta at a single cardiac phase, it is not feasible to solve for both unknowns simultaneously using noninvasive methods. Fortunately, for statically determinate structures, the deformed geometry and boundary conditions alone can be sufficient to determine the structural stress distribution in the deformed configuration. The aortic wall can be treated as approximately statically determinate because its thickness is relatively small compared to its diameter. Regardless of the constitutive behavior, the internal forces (i.e., structural stresses) of the aortic wall must balance the same external load (i.e., intraluminal blood pressure). This equilibrium can be approximated locally based on the aortic wall geometry and the applied pressure (Joldes et al. 2016), which makes the resulting structural stress only weakly dependent on the specific material properties of the wall.

The forward penalty approach (Joldes et al. 2016) leverages the static determinacy principle by simplifying computation: an artificially stiff material is assigned as a penalty treatment, which leads to minimal deformation (e.g., a stiffness of 5 × 10^5^ kPa typically results in displacements on the order of 10⁻^3^ mm under physiological pressure). In this approach, the CT-derived deformed aortic geometry is used as the input ‘undeformed’ shape. Because the induced deformation is negligible, the resulting ‘deformed’ configuration remains nearly identical. Due to static determinacy, i.e., the computed structural stress field is approximately independent of the constitutive relation, the structural wall stress computed under the artificial stiffness can closely approximate the ‘true’ stress field under patient-specific constitutive behavior, despite the patient-specific constitutive behavior being unknown with noninvasive means.

**Fig. 5:**
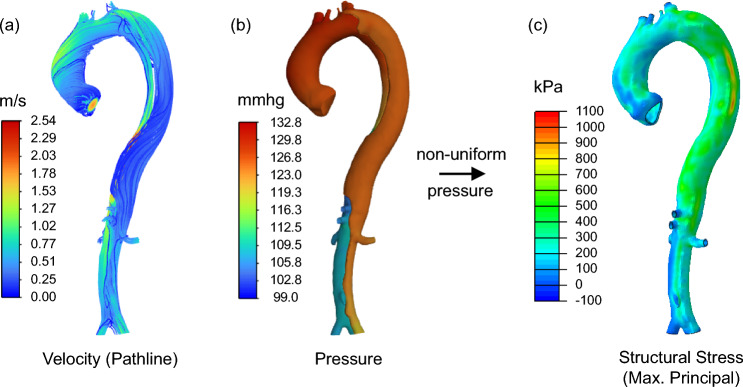
Forward penalty method workflow for structural stress computation: **a** flow pathlines color-coded with velocity; **b** pressure contour; **c** structural wall stress distribution computed by enforcing an artificially stiff material as penalty treatment to the aortic wall

As depicted in Fig. 5, forward penalty structural stress computations were conducted utilizing the CTA at initial diagnosis of each patient, involving the following steps: (1) Patient-specific computational fluid dynamics (CFD) simulations were performed in ANSYS Fluent: For patients P2, P3, P4, P6, and P7, a turbulent 1/7 power law velocity profile (Štigler 2014), with maximal velocity derived from patient-specific echocardiographic measurements of peak aortic valve velocity, was used as the steady-state inlet flow condition for the ascending aorta. For patients P1, P5, P8, and P9, where echocardiography data were unavailable, the same 1/7 power law velocity profile with a maximal velocity was applied to achieve a flow rate of 25 L/min, which represents a population-averaged physiological flow rate during peak systole (approximately 417 mL/s) (Bonfanti et al. 2017). The total vascular resistance for each patient was determined by dividing the patient-specific systolic blood pressure obtained during the initial hospital admission by the corresponding flow rate. Based on this total resistance, Windkessel boundary conditions were imposed at each outlet of the descending aortic branches by allocating the total vascular resistance proportionally to each branch’s cross-sectional area, following Murray’s law (Fevola et al. 2021). With this setup, the simulated systolic blood pressure at the aortic arch generally matched the patient’s clinical measurement in magnitude. Due to the complexity of patient-specific anatomies, in 7 of 9 patients (P1, P3, P5, P6, P7, P8, and P9), the calculated total resistance initially resulted in a pressure deviation exceeding 2% MAPE compared to the clinically measured systolic blood pressure. In these cases, total resistance was fine-tuned to reduce the deviation to within 2% MAPE, with outlet resistances again distributed according to Murray’s law. (2) The nonuniform blood pressure distribution in the true and false lumen walls was extracted from the CFD simulation and mapped to the solid domain mesh using an in-house 3D interpolation code. (3) Subsequently, maximum principal structural stress distributions in the true and false lumen walls, as well as the dissection flap, were derived from structural finite element analysis (FEA) in Abaqus using the spatially varying pressure distribution computed in the CFD simulation as the loading condition. Using the principle of static determinacy of the aortic wall (Liu et al. 2019b), structural wall stress was computed by enforcing an artificially stiff material (Young’s modulus 5 × 10^5^ kPa) as penalty treatment to the aortic wall, ensuring that the displacement remains negligibly small (on the order of 10^–3^ mm), which eliminated the need for patient-specific material properties or realistic aortic wall deformations. For cases with intraluminal thrombus, a Young’s modulus of 2.5 × 10^4^ kPa was assigned to the thrombus, corresponding to a wall-to-thrombus artificial stiffness ratio of 1:20, following Joldes et al. 2016. Assigning different artificial stiffness values based on the actual tissue stiffness ratio enables a physiologically realistic structural stress distribution.

**Fig. 6:**
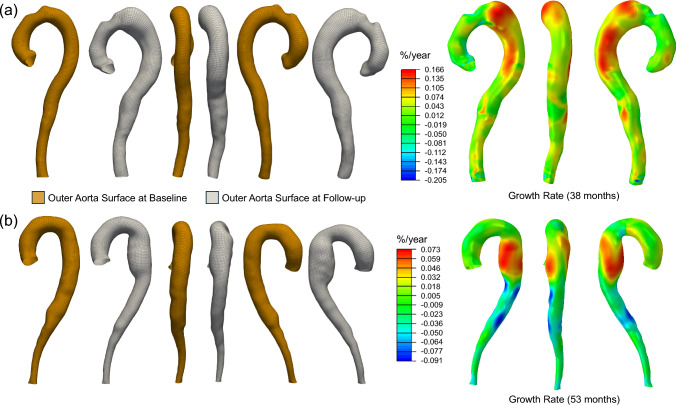
Outer aortic surface meshes at baseline and follow-up for two representative TBAD patients, along with the corresponding aortic growth rate heatmaps. **a** P3; **b** P9

TBAD anatomy may be distinct from that of a healthy or aneurysmal aorta due to the presence of the dissection flap. To validate the forward penalty structural stress computation approach for TBAD geometries, we compared the structural stress field obtained using the forward penalty method with that computed via a fully coupled steady-state FSI approach. The MAPE of the two structural stress fields is 7.57%. The details of this validation are provided in Appendix. To evaluate the impact of regional material property differences on static determinacy, a sensitivity analysis was conducted by varying the artificial stiffness of the true lumen, false lumen, and dissection flap by  ± 50% relative to the value of *E* = 5 × 10^5^ kPa. The results, presented in Appendix (Fig. 6), indicate that the computed structural stress field is relatively insensitive to such variations.

### Quantification of aortic growth rate

For each patient, the follow-up TBAD geometry was segmented from most-recent follow-up CTA and meshed with 2D elements, following the same procedure described in Sect. 2.2. In this study, we computed the aortic growth rate at every node of the TBAD meshes using the initial and most recent follow-up CTA scans, which allowed visualization of the growth rate as a heatmap distribution. To generate this heatmap, the outer aorta surface of the baseline TBAD geometry at initial diagnosis was re-meshed by using a mesh parameterization approach (Liang et al. 2017). Thus, this surface mesh consists only of approximately equal-sized quadrilateral elements and is topologically equivalent to the lateral surface of a cylinder. It consists of 50 nodes along each circumference and 200 circumferential layers along the axial direction, forming a structured mesh. The local aortic coordinate system (radial, circumferential, and axial directions) was established following our previous work (Liu et al. 2019a; Liang et al. 2017). Specifically, the center point of each of the 200 layers was determined by averaging the coordinates of the 50 surrounding nodes. For each node on the structured aortic surface mesh, the radial direction was defined as the outward-pointing surface normal at that location. The axial direction was defined as the vector connecting the center points of the current and subsequent layers. The circumferential direction was then computed as the orthogonal cross-product of the radial and axial vectors. The baseline and follow-up meshes were then pre-aligned using the iterative closest point (ICP) algorithm (Sharp et al. 2002) to remove rigid body motions. The ICP algorithm begins with an initial guess of the transformation, defined by a rotation matrix ${R}_{0}$​ and translation vector ${t}_{0}$, such that the initial transformed point cloud is ${P}_{0}={R}_{0}P+{t}_{0}$​. At each iteration $k$, for every point ${p}_{i,k}$ (where $i=1,\dots ,N$) in the source point cloud, the algorithm identifies the closest corresponding point ${q}_{i,k}$​ in the target point cloud. The transformation for the next iteration, ${R}_{k+1}$ and ${t}_{k+1}$​, is obtained by minimizing the following objective function:

1 $${R}_{k+1},{t}_{k+1}=\underset{R,t}{\mathrm{argmin}}\sum_{i=1}^{N}{\Vert \left(R{p}_{i,k}+t\right)-{q}_{i,k}\Vert }^{2}$$

The optimal $R$ and $t$ are computed using singular value decomposition (SVD). The algorithm terminates when the average change in estimated rigid transformations over the three most recent iterations falls below specified tolerance thresholds. In this study, the rotation and translation tolerances were set to 0.0001 and 0.0005, respectively. The initial guess ${R}_{0}$​ and ${t}_{0}$ was set to identity rotation and zero translation. If visual inspection revealed obvious misalignment, the initial rotation ${R}_{0}$ was manually adjusted, for example, by applying a 30-degree rotation about the x-, y-, or z-axis, or a combination thereof, until a satisfactory alignment was achieved.

Subsequently, the baseline geometry was morphed to match the follow-up geometry through a large deformation diffeomorphic metric mapping framework (Durrleman et al. 2014) implemented in the Deformetrica software (Bône et al. 2018). Specifically, Deformetrica parametrizes diffeomorphisms ${\Phi }_{\lambda ,\mu }$ using a set of control points $\lambda =[{\lambda }_{1},.., {\lambda }_{m}]$ and corresponding momentum vectors $\mu =[{\mu }_{1},.., {\mu }_{m}]$. In nonrigid registration tasks, the goal is to find a diffeomorphism ${\Phi }_{\lambda ,\mu }$ that deforms a template mesh $T$ (represented by edge centers ${c}^{(T)}=[{c}_{1}^{(T)},.., {c}_{{N}^{(T)}}^{(T)}]$ and their normals ${n}^{(T)}=[{n}_{1}^{(T)},.., {n}_{{N}^{(T)}}^{(T)}]$) to match a target mesh $S$ (represented by edge centers ${c}^{(S)}=[{c}_{1}^{(S)},.., {c}_{{N}^{(S)}}^{(S)}]$ and their normals ${n}^{(S)}=[{n}_{1}^{(S)},.., {n}_{{N}^{(S)}}^{(S)}]$). The following cost function is minimized:

2 $$cost\left(\lambda ,\mu \right)=\frac{1}{{\sigma }^{2}}\sum_{i}\sum_{j}{K}_{W}\left({c}_{i}^{{\Phi }_{\lambda ,\mu }(T)},{c}_{j}^{(S)}\right)\frac{{\left({\left({n}_{i}^{{\Phi }_{\lambda ,\mu }(T)}\right)}^{T}{n}_{j}^{(S)}\right)}^{2}}{\Vert {n}_{i}^{{\Phi }_{\lambda ,\mu }(T)}\Vert \Vert {n}_{j}^{\left(S\right)}\Vert }+\sum_{k}\sum_{l}{\left({\mu }_{k}\right)}^{T}{K}_{R}\left({\lambda }_{k},{\lambda }_{l}\right){\mu }_{l}$$

where the first term in Eq. (2) represents the data attachment, which measures how well the deformed template matches the target via a varifold-based distance that incorporates spatial location and orientation with a kernel ${K}_{W}$. The second term serves as a regularization that penalizes the kinetic energy of the deformation using a kernel function ${K}_{R}$ over the control points. The relative importance of those two terms is controlled by the parameter $\sigma$. In this study, $\sigma$ was set to 1; both the kernel widths in ${K}_{W}$ and ${K}_{R}$ were set to be 20, following the calibration process suggested by Williams et al. 2022. These values balance accuracy in shape matching with smoothness in the resulting deformation. The nonrigid registration produced a follow-up structured mesh with node-to-node correspondence to the baseline structured mesh.

This nonrigid registration method has been validated through benchmark testing for establishing point-to-point mesh correspondence (Goparaju et al. 2022). A similar approach, known as registration-based aortic growth mapping (Dux-Santoy et al. 2022; Merton et al. 2024a, b; Teixido-Tura et al. 2024) or vascular deformation mapping (Burris et al. 2022, 2017) or, more generally, registration-based aortic growth mapping, has also been validated against manual measurements (Burris et al. 2022; Dux-Santoy et al. 2022; Merton et al. 2024a, b; Teixido-Tura et al. 2024).

Following the nonrigid registration, the displacement field derived from pointwise correspondence between the baseline and follow-up TBAD geometries was applied as a displacement boundary condition in Abaqus to compute the 3D logarithmic strain field representing aortic growth. The aortic growth rate was then determined by dividing the circumferential strain by the number of years between the baseline and follow-up CTA. The workflow for quantifying the aortic growth rate is illustrated in Fig. 6. This work presents growth rate heatmaps in %/year, as mm/year is not a meaningful metric at localized spatial points. To improve clinical interpretability, the spatially averaged aortic diameter growth rate (in mm/year) was computed for each patient using the structured surface meshes at baseline and follow-up. For each layer of the baseline mesh, the local aortic diameter was calculated by first determining the center point of the layer, then averaging the distances from this center to the 50 surrounding nodes. The descending aorta region was defined using the point just distal to the left subclavian artery as the proximal landmark. The spatially averaged diameter was then calculated by averaging layers starting from the one closest to this landmark up to layer 200, which corresponds to the aortic bifurcation. For patients P1 ~ P9, the closest layers to this landmark were 42, 44, 49, 49, 49, 49, 49, 41, and 42, respectively. The resulting descending aorta region for each patient is shown in Figure 17. The same procedure was applied to the follow-up scan. The aortic diameter growth rate was then calculated as the difference between the follow-up and baseline spatially averaged diameters, divided by the time interval (in years) between the two scans.

**Fig. 7:**
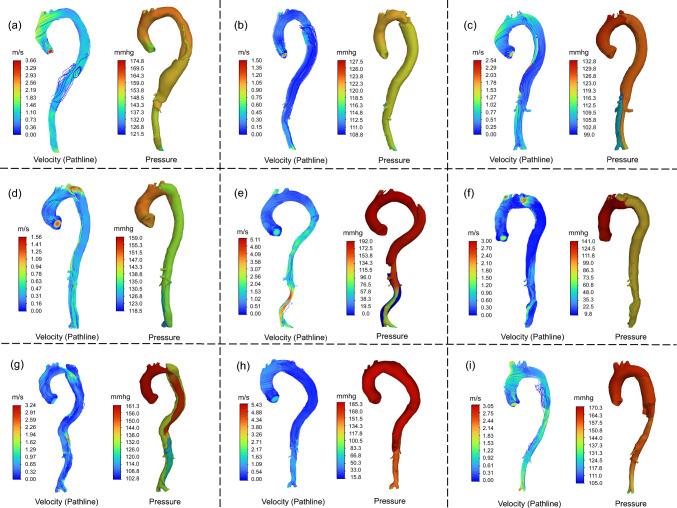
Pressure distribution and pathline results of the TBAD patients. The pathlines were color-coded with the velocity of blood flow. **a** P1; **b** P2; **c** P3; **d** P4; **e** P5; **f** P6; **g** P7; **h** P8; **i** P9

### Statistical analysis

To assess the 3D spatial correlation between the distribution of structural wall stress and the heatmap of aortic growth rate, we first aligned the structural wall stress and growth rate distribution data and established point-to-point correspondence, i.e., the subsequent spatial correlation analysis requires that a spatial point on the structural stress distribution is aligned with the same spatial point on the growth rate heatmap. To achieve this, the structural stress distribution was registered onto the structured mesh where growth rate data were stored: because the structural wall stress distribution was originally computed on the solid domain finite element mesh, while the aortic growth rate heatmap was computed on the structured quadrilateral surface mesh. To enable pointwise correspondence between the structural stress and growth rate, rigid body motions between the finite element mesh and structured surface mesh were removed via the same ICP (Sharp et al. 2002) registration procedure described in Sect. 2.3, using identical tolerance parameters. Subsequently, for each node on the structured surface mesh, the closest node on the finite element mesh was identified, and the corresponding structural stress value was assigned to the structured surface node. This process was repeated for all nodes on the structured surface. As a result, the structural stress distribution was registered to the structured surface, and the structural stress and growth data share the same 3D coordinates. To enhance data analysis and visualization, the following sampling method was employed. Using the structured mesh from the growth rate analysis (50 nodes per circumference and 200 circumferential layers along the axial direction), each patient’s outer aortic surface was initially divided into 50 approximately equal-sized regions, with each region consisting of 4 circumferential layers. In this partitioning, the start of the 13th region (layer 49) was intended to align with the point just distal to the left subclavian artery, which was achieved for patients P3, P4, P5, P6, and P7. For P1, P2, P8, and P9; however, the closest layers to this landmark were 42, 44, 41, and 42, respectively. To ensure consistency across the cohort, the partitioning was manually adjusted so that the start of the 13th region consistently aligned with the landmark. After adjustment, the partitioning remained approximately uniform: in the ascending aorta/aortic arch (regions 1 ~ 12), each region contained a similar number of circumferential layers, and in the descending aorta (regions 13 ~ 50), the regions likewise contained approximately equal numbers of circumferential layers. Based on this partitioning, the descending aorta, from the left subclavian artery to the aortic bifurcation, consists of 38 regions. Subsequently, the averaged maximum principal structural stress and aortic growth rate can be calculated for each region, which resulted in 38 stress–growth rate data points for each patient. This data sampling method allowed for the quantification and visualization of the structural stress–growth rate spatial correlation.

To account for inter-patient variability, linear mixed-effects regression analysis (Oberg and Mahoney 2007) was then conducted to investigate the relationship between structural wall stress ${\sigma }_{im}$ and aortic growth rate ${GR}_{im}$, where $m=\mathrm{1,2},\dots ,9$ denotes patient ID, and $i=\mathrm{1,2},\dots ,342$ indexes data points (38 regions each patient, totaling 342 data points across 9 patients). In the linear mixed-effects model, the growth rate can be modeled as:

3 $${GR}_{im}={\beta }_{0}+{\beta }_{1}{\sigma }_{im}+{b}_{0m}+{b}_{1m}{\sigma }_{im}+{\varepsilon }_{im}$$

where the slope and intercept are separated into fixed-effects (slope ${\beta }_{1}$ and intercept ${\beta }_{0}$) and random-effects (slopes ${b}_{1m}$ and intercept ${b}_{0m}$). The fixed-effects (${\beta }_{1}$ and ${\beta }_{0}$) remain constant across all patients, which characterize the overall association between structural stress and growth rate across the entire patient population ($n=9$); the random-effects (${b}_{1m}$ and ${b}_{0m}$) are adjusted to fit the individual structural stress–growth rate relationships for each patient that are not fully explained by the fixed effects, allowing both the intercept and slope to vary by patient and thereby accounting for inter-patient variations. The residual term ${\varepsilon }_{im}$ captures unexplained response at each data point. The significance of the structural stress–growth rate association was determined by performing an F-test on the fixed-effect slope ${\beta }_{1}$ with the null hypothesis being ${\beta }_{1}=0$. A *p* value smaller than 0.05 was considered statistically significant to reject the null hypothesis and indicate a nonzero fixed-effect slope ${\beta }_{1}$. Additionally, we tested the inclusion of a quadratic fixed-effect term (${\beta }_{2}{\sigma }_{im}^{2}$) to evaluate its potential impact on model performance using Akaike information criterion (AIC) and Bayesian information criterion (BIC). However, additional nonlinear terms may not be necessary, as they could improve model fit without necessarily improving generalizability to new data. To evaluate whether a specific random-effect term (${b}_{1m}$ or ${b}_{0m}$) in Eq. (3) was potentially redundant and quantify inter-patient variability, we estimated the standard deviation and its 95% confidence interval (CI) of each term. If the CI did not include zero, the corresponding random-effect was considered statistically significant and retained in the model. For each individual patient, the structural stress–growth rate correlation was further assessed using Pearson’s correlation coefficient and its associated *p* value.

For comparison with structural stress performance, we also used systolic WSS and systolic blood pressure as predictors in linear mixed-effects regression analyses. To establish pointwise correspondence between WSS, blood pressure, and growth rate, the WSS and blood pressure fields from CFD were registered to the growth-rate heatmap following the same steps described above for structural stress. First, ICP registration was applied to remove rigid body motions between the CFD mesh and the structured surface mesh. Next, each node on the structured surface mesh was assigned the WSS and blood pressure values from its closest node on the CFD mesh. Following the same partitioning process that divided the descending aorta of the structured surface mesh into 38 regions, the WSS, blood pressure, and growth rate values were averaged within each region, resulting in 38 paired data points for WSS-growth rate or blood pressure-growth rate analyses for each patient. For WSS, the model was:

4 $${GR}_{im}={\beta }_{0}+{\beta }_{1}{WSS}_{im}+{b}_{0m}+{b}_{1m}{WSS}_{im}+{\varepsilon }_{im}$$

where ${WSS}_{im}$ is the average systolic WSS value in a region (global point index $i$) of patient $m$. Similarly, for blood pressure, the model was:

5 $${GR}_{im}={\beta }_{0}+{\beta }_{1}{BP}_{im}+{b}_{0m}+{b}_{1m}{BP}_{im}+{\varepsilon }_{im}$$

where ${BP}_{im}$ is the average systolic blood pressure value in a region (global point index $i$) of patient $m$. Statistical testing for the fixed- and random-effects slopes (${\beta }_{1}$ and ${b}_{1m}$) followed the same procedures as in the structural stress–growth rate analysis.

To mitigate the potential impact of nonlinear aortic growth caused by varying follow-up intervals across the patient cohort, we performed an additional linear mixed-effects regression including follow-up time as a fixed-effect covariate.

To further investigate the potential associations between local thrombus and structural stress, as well as between local thrombus and aortic growth rate, we performed additional linear mixed-effects analyses. For each node of the structured mesh where structural stress and growth rate data were stored, we calculated the distance to the nearest thrombus node on the finite element mesh after ICP registration. If the distance was less than the calculated radius of the node, local thrombus was considered present at the corresponding point; otherwise, it was considered absent. Consequently, local thrombus was modeled as a binary presence (1)/absence (0) variable on the structured surface mesh for each patient, with a one-to-one correspondence to structural stress and aortic growth rate. Following the partitioning process described above, the linear mixed-effects models assessed the role of thrombus using 38 regions, each constructed by averaging the thrombus values. Regions with an average thrombus value > 0.5 were considered thrombus-occupied (${Tb}_{im}$ = 1). Otherwise, ${Tb}_{im}$ = 0. This resulted in 38 pairs of thrombus-structural stress or thrombus-growth rate data points for each patient. For structural stress, the model was:

6 $${\sigma }_{im}={\beta }_{0}+{\beta }_{1}{Tb}_{im}+{b}_{0m}+{b}_{1m}{Tb}_{im}+{\varepsilon }_{im}$$

Similarly, for aortic growth rate, the model was:

7 $${GR}_{im}={\beta }_{0}+{\beta }_{1}{Tb}_{im}+{b}_{0m}+{b}_{1m}{Tb}_{im}+{\varepsilon }_{im}$$

Hypothesis testing for the fixed- and random-effects slopes (${\beta }_{1}$ and ${b}_{1m}$) was conducted in the same manner as described previously for structural stress as a predictor.

## Results

### Study sample

The demographic information of the patients involved in this study is summarized in Table 1. Among the 9 patients analyzed for outcomes, the median follow-up period was 3.18 years. Spatially averaged growth rates of the descending aorta were calculated for each patient using the method described in Sect. 2.4 (see Table 2), resulting in a median spatially averaged descending aorta growth rate of 1.84 mm/year. Table 1 also presents the median and interquartile ranges of demographic variables such as age, sex, and the number of visceral vessels originating from the true and false lumens.

**Table 1:** Patient demographics, comorbidities, and dissection characteristics of the study population

Age at the time of diagnosis (years)	62 [49.5, 67.5]
Sex (% male)	44.44
Height (m)	167.7 [159.38, 179.08]
Weight (kg)	88.7 [77.18, 98.13]
Systolic brachial blood pressure (mmHg)	156 [133.75, 171.75]
Diastolic brachial blood pressure (mmHg)	80 [62.5, 94]
Beta-blocker (%)	22.22
Hypertension (%)	88.89
Diabetes mellitus (%)	0
Stroke or transient ischemic attack (%)	0
Dyslipidemia (%)	0
End-stage renal disease (%)	0
Smoking (%)	44.44
Congestive heart failure (%)	0
Connective tissue disorder (%)	0
False lumen thrombosis (%)	66.67
Number of arteries arising from true lumen	3[2, 4.5]
Number of arteries arising from false lumen	2 [0.5, 4]
Follow-up period (years)	3.18 [1.19, 4.83]
Spatially averaged aortic growth rate (mm/year)	1.84 [0.32, 2.32]

The number of arteries includes celiac, superior mesenteric, inferior mesenteric, left renal, and right renal arteries. For numerical variables, the median, 25th and 75th percentiles are reported. Percentage is reported for dichotomic variables

**Table 2:** Baseline and follow-up CTA dates, time intervals, spatial resolutions, and spatially averaged aortic diameter growth rates for the TBAD patients

Patient ID	Date of baseline CTA (mm/dd/yyyy)	Date of follow-up CTA (mm/dd/yyyy)	Duration (months)	In-plane resolution (mm)	Axial resolution (mm)	Spatially averaged aortic growth rate (mm/year)
1	12/16/2015	08/18/2016	8	0.826 × 0.826	1.000	− 5.50
2	03/19/2015	11/30/2016	21	0.715 × 0.715	1.000	0.50
3	04/01/2008	06/15/2011	38	0.895 × 0.895	1.250	2.43
4	04/06/2013	10/04/2016	42	0.873 × 0.873	1.250	2.28
5	03/25/2016	10/11/2018	30	0.721 × 0.721	1.000	1.92
6	03/15/2010	08/25/2017	91	0.742 × 0.742	1.500	1.83
7	04/10/2023	07/06/2023	3	0.890 × 0.890	1.000	12.50
8	01/15/2009	03/22/2012	38	0.586 × 0.586	0.600	1.02
9	10/20/2011	03/24/2016	53	0.742 × 0.742	1.500	− 0.20

The CTA images had an average in-plane resolution of 0.777 × 0.777 mm and an average axial resolution of 1.122 mm. The baseline and follow-up CTA dates, scan intervals, and resolutions for each patient are summarized in Table 2. False lumen thrombus was present in the initial baseline CTAs of patients P1, P2, P5, P6, P7, and P9. Patients P3, P4, and P8 had patent false lumens without signs of thrombosis, and therefore thrombosis was not included in the simulations for these cases.

### Hemodynamics

Table 3 summarizes the fine-tuning adjustments to total resistance for each patient, along with flow rates and simulated versus clinically measured systolic blood pressures. For all patients, the simulated systolic blood pressure at the aortic arch closely matched the clinical measurements obtained at initial hospital admission, with a MAPE of less than 2%. Hemodynamic outcomes, including pressure distribution and pathlines of TBAD blood flow, were computed from the forward penalty analyses, as depicted in Fig. 7. The pathline results were color-coded according to the velocity of blood flow. Blood pressure is proportional to structural wall stress in an idealized cylinder, according to the law of Laplace (Ban et al. 2023; Elefteriades et al. 2021). Utilizing Windkessel outlets, the simulated systolic blood pressure in the ascending aorta was consistent with clinically measured systolic blood pressure from the patient’s right arm. Pressure differences between the true and false lumens stemmed from unique geometric characteristics of TBAD geometries, such as the size and location of the primary intimal tear and the presence of intraluminal thrombosis.

**Table 3:** Brachial systolic blood pressure (BP) measurements obtained during the initial hospital admission and the averaged systolic pressure in aortic arch region obtained from CFD simulations of the TBAD patients

Patient ID	Measured systolic BP at initial hospital admission (mmHg)	Simulated systolic BP (mmHg)	Systolic flowrate (L/min)	Calculated total resistance (mmHg min/L)	Tuned total resistance (mmHg min/L)	Resistance adjustment (%)
1	159	159	25.00	6.36	5.40	−15.00
2	119	121	8.89	13.39	13.39	0.00
3	127	128	24.18	5.25	4.78	−9.08
4	147	146	26.64	5.52	5.52	0.00
5	191	192	25.00	7.64	4.80	−37.16
6	136	135	15.06	9.03	6.50	−28.08
7	156	158	26.70	5.84	9.83	68.13
8	183	185	25.00	7.32	5.66	−22.55
9	168	168	25.00	6.72	5.44	−19.09

Total resistances in the simulations were adjusted until the simulated pressures matched the measured values within a 2% mean absolute percentage error (MAPE)

For each patient, we first computed the spatially averaged pressures in the true and false lumens from CFD simulations. Across the 9 patients, the mean spatially averaged pressure was 137.72 mmHg in the true lumen and 132.04 mmHg in the false lumen (median [interquartile range]: 140.77 [119.49, 155.74] mmHg vs. 135.87 [113.41, 156.07] mmHg, respectively). A paired-sample t test yielded a *p* value of 0.4285, indicating no statistically significant difference in spatially averaged pressure between the true and false lumens. The false lumen exhibited a more uniform pressure distribution, which may be attributed to reduced flow resulting from the limited size of entry tears. This restriction can lower peak pressures and raise minimum pressures, thereby flattening the overall pressure profile and reducing spatial pressure variation within the false lumen. Combined with the pressure contour results, these findings suggest that pressure distributions between the true and false lumens are highly patient-specific.

### Contours of structural stress and aortic growth rate

**Fig. 8:**
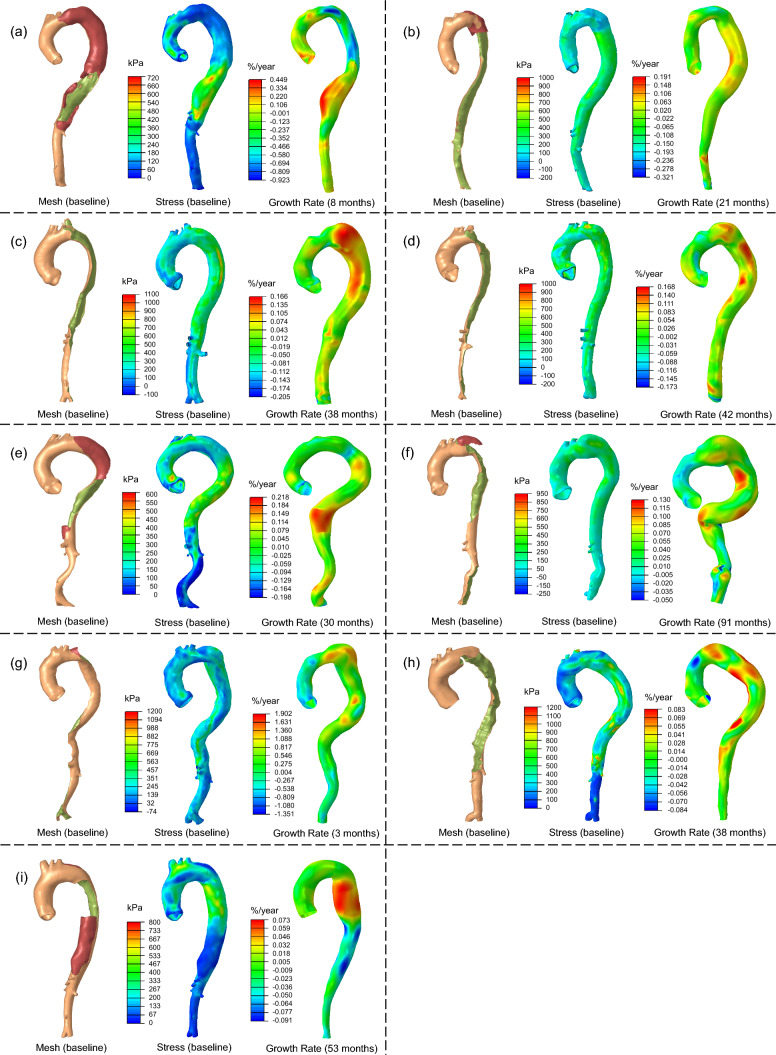
Aortic geometries and structural wall stress distributions at baseline diagnosis, along with corresponding heatmaps of aortic growth rates for each patient: **a** P1; **b** P2; **c** P3; **d** P4; **e** P5; **f** P6; **g** P7; **h** P8; **i** P9. The aortic growth rate heatmaps are presented on the structured surface mesh of the outer aorta at follow-up

Structural wall stress fields computed based on the initial baseline CT scans were compared to the heatmap of the aortic growth rate displayed on the follow-up geometries, as depicted in Fig. 8. Alternatively, the aortic growth heatmaps were also displayed on the baseline geometries in Fig. 18. Results from the analysis of the 9 patients revealed a general correlation between structural stress and growth rate: aortic segments experiencing high structural wall stress demonstrated rapid aortic growth. This correlation was particularly evident in patients P1, P3, P5, P7, P8, and P9. For instance, the distal descending aorta of P1 exhibited the highest structural wall stress in the baseline geometry, and this same region experienced rapid aortic growth (~ 30%/year) over the follow-up period (8 months).

In four patients (P1, P2, P5 and P9), negative aortic growth rates were observed in segments of descending aorta due to a reduction in aortic diameter in the follow-up scan compared to the baseline scan. We hypothesize that the diameter reduction was driven by thrombus formation and the subsequent reduction in structural stress, which promoted favorable remodeling and healing of the dissection. Comparison between TBAD geometries and structural stress distributions (Fig. 8) revealed that regions with thrombosed false lumens exhibited much lower structural wall stress compared to other regions exposed to high blood pressure. This finding suggests that thrombus may serve a protective role against high structural wall stress. In Sect. 3.5, we quantitatively investigate the predictive role of local thrombus on structural stress and aortic growth rate.

In contrast to the high structural stress observed in the true and false lumens, the dissection flap experienced significantly lower structural stress levels, typically below 50 kPa. This is attributed to the fact that the dissection flap is subjected to pressures from both the true and false lumens.

### Quantification of spatial correlation between structural stress and aortic growth rate

**Fig. 9:**
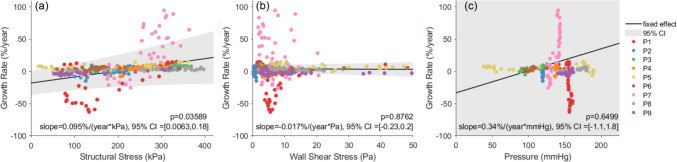
**a** Linear mixed-effects regression analysis demonstrates a significant fixed-effect association between structural wall stress at initial baseline diagnosis and aortic growth rate (*p* = 0.039). The same analysis indicated insignificant fixed-effect associations using wall shear stress (WSS) **b** and blood pressure **c** distributions. CI: confidence interval

Linear mixed-effects regression analysis was performed which revealed a positive fixed-effect association between structural wall stress and growth rate considering all 9 patients. As shown in Fig. 9, F-test indicated that the fixed-effect slope ${\beta }_{1}$ was statistically significant (*p* = 0.036) to reject the null hypothesis that ${\beta }_{1}$ is zero. The estimated ${\beta }_{1}$ in the mixed-effects model was 0.095%/(year·kPa) with a 95% CI of [0.0063, 0.18]%/(year·kPa). Including random effects improves the overall estimate of the fixed-effect slope (${\beta }_{1}$) by explicitly accounting for inter-patient variability. A fixed-effects-only model yielded ${\beta }_{1}$ = 0.074%/(year kPa) with a 95% CI of [0.055, 0.094]%/(year·kPa) and *p* < 0.0001, but this *p* value may be overly optimistic when inter-patient variability is not considered. For comparison, the systolic WSS and systolic blood pressure were also tested as predictors in linear mixed-effects models. WSS led to a near-zero, negative fixed-effect ${\beta }_{1}$ (-0.017%/(year·Pa)), while pressure resulted in a positive fixed-effect ${\beta }_{1}$ (0.34%/(year·mmHg)). However, the 95% CI for pressure was wide ([− 1.14, 1.83] %/(year·mmHg)) due to highly patient specific spatial pressure distributions. The results indicated that the fixed-effect associations were not statistically significant for WSS (*p* = 0.88) or blood pressure (*p* = 0.65). We also tested the inclusion of a quadratic fixed-effect term (${\beta }_{2}{\sigma }_{im}^{2}$) in Eq. (3). The results showed a slight reduction in AIC from 2749.0 to 2741.2, and a marginal reduction in BIC from 2768.2 to 2764.2, suggesting that the second-order term did not provide substantial improvement to model performance. To account for the potential impact of nonlinear aortic growth, we performed an additional linear mixed-effects regression analysis including follow-up time as a fixed-effect covariate. Incorporating follow-up time did not significantly alter the results: the *p* values for the fixed-effect associations with structural stress (*p* = 0.40), wall shear stress (*p* = 0.88), and pressure (*p* = 0.65) remained largely unchanged.

**Fig. 10:**
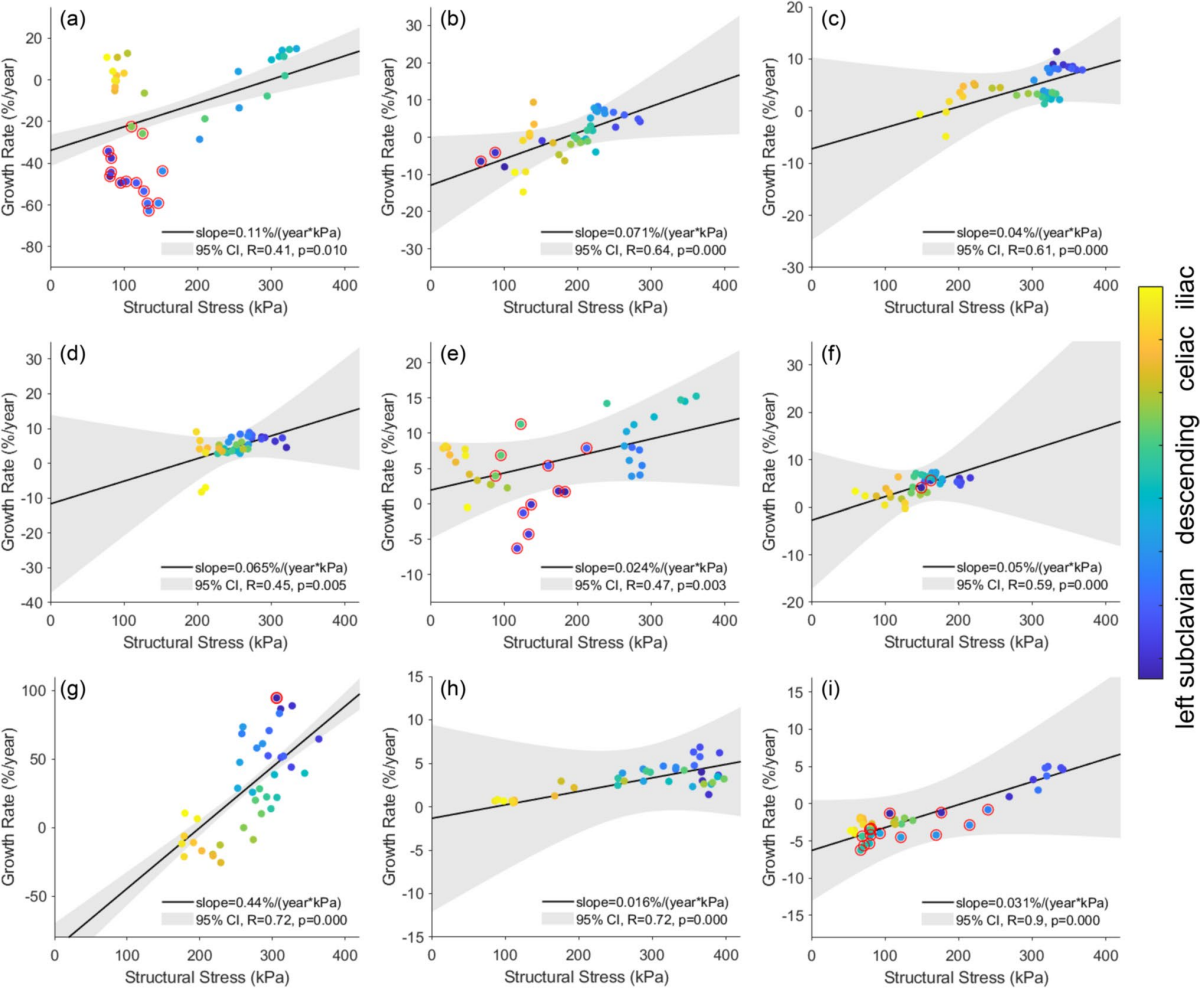
Structural wall stress distribution at initial baseline diagnosis and aortic growth rate of individual patients. **a** P1; **b** P2; **c** P3; **d** P4; **e** P5; **f** P6; **g** P7; **h** P8; **i** P9. The structural stress–growth rate data points are color-coded by spatial location from the proximal to the distal aorta. Red circles indicate regions with intraluminal thrombus. The regression lines were obtained by using both fixed- and random-effects in the linear mixed-effects model. CI: confidence interval; R, *p*: Pearson correlation coefficient and *p* value

**Fig. 17:**
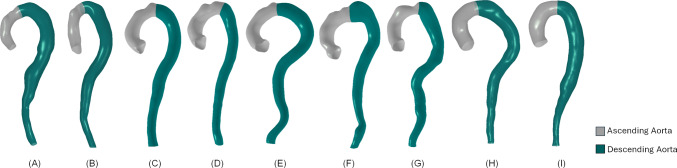
Descending aorta regions of the TBAD patients ((A–I) correspond to P1 ~ P9). The ascending aorta is shown in gray, and the descending aorta is highlighted in green

**Fig. 18:**
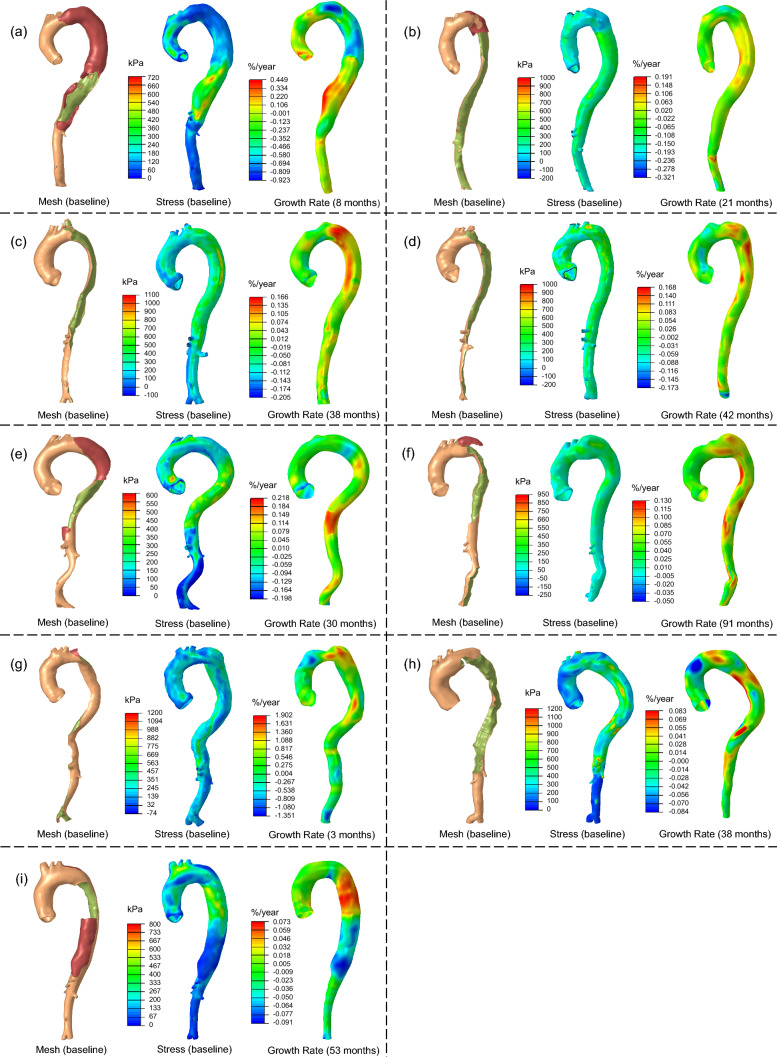
Aortic geometries and structural wall stress distributions at baseline diagnosis, along with corresponding heatmaps of aortic growth rates for each patient: **a** P1; **b** P2; **c** P3; **d** P4; **e** P5; **f** P6; **g** P7; **h** P8; **i** P9. The aortic growth rate heatmaps are presented on the structured surface mesh of the outer aorta at baseline

Figure 10 summarizes the individual correlations between structural wall stress and growth rate for the 9 patients. Data points are color-coded by spatial location from the proximal to the distal aorta. The results align with the observations in Fig. 8. For instance, in Fig. 10a, the distal descending aorta (‘cyan’ dots) exhibits the most rapid aortic expansion and highest structural stress, whereas the proximal descending aorta (‘dark blue’ dots) demonstrates a negative growth rate and much lower structural wall stress. The *p* values for Pearson correlation coefficients were significant for all 9 patients. The standard deviations of the random-effect slopes (${b}_{1m}$) and intercepts (${b}_{0m}$) were estimated to be 0.13%/(year·kPa) with a 95% confidence interval (CI) of [0.079, 0.22]%/(year·kPa), and 27.80% with a 95% CI of [16.45, 46.97]%, respectively. The results indicate substantial inter-patient variability and support the inclusion of random effects in Eq. (3). They are also consistent with the observation that regions experiencing high structural stress are correlated with a faster aortic growth. Individual correlations between WSS, pressure and growth rate are summarized in Appendix.

### Quantification of spatial correlation between thrombus, structural stress, and aortic growth rate

For patients with thrombosis (P1, P2, P5, P6, P7, and P9), the red-circled data in Fig. 10 indicate that regions covered by thrombus generally exhibit lower structural stress compared to neighboring regions of similar color, which is associated with slower aortic growth.

To quantify the association between thrombus and structural stress, we performed linear mixed-effects analyses. Local thrombus showed a significant fixed-effect association with structural stress both without random effects (${\beta }_{1}$= − 99.46 kPa, 95% CI [− 127.28, − 71.64] kPa, *p* < 0.0001), where ${\beta }_{1}$ represents the average reduction of structural stress in the presence of local thrombus, and with random effects (${\beta }_{1}$= − 55.16 kPa, 95% CI [− 81.53, − 28.79], *p* < 0.0001). The standard deviation of the random-effect slopes ${b}_{1m}$ could not be estimated using MATLAB’s ‘fminunc’ optimization algorithm, which suggests negligible variability across patients. However, the random-effect intercepts (${b}_{0m}$) had a significant standard deviation of 52.77 kPa (95% CI [48.95, 56.89]), which shifted the estimate of ${\beta }_{1}$ upward in the mixed model. These results support the observation that thrombus is associated with locally reduced structural stress, consistent with Fig. 8, and suggest a protective role in reducing wall stress.

We also tested the association between local thrombus and aortic growth rate. Considering only fixed effects, the association was significant (${\beta }_{1}$= − 19.15%/year, 95% CI [− 24.58, − 13.71]%/year, *p* < 0.0001), where ${\beta }_{1}$ indicates the average reduction in growth rate where thrombus is present. However, when random effects were included, the fixed-effect association was not significant (${\beta }_{1}$= − 0.37%/year, 95% CI [− 26.74, 25.99]%/year, *p* = 0. 9777). The standard deviation of the random-effect slopes (${b}_{1m}$) was 31.90%/year (95% CI [17.09, 59.55]%/year), which suggests considerable inter-patient variability that may obscure the thrombus–growth relationship in a mixed-effects framework.

## Discussion

### Forward penalty structural stress analysis as a potential predictive tool

In this study, we investigated the relationship between the structural wall stress distribution and aortic growth rate in acute uncomplicated TBAD using a forward penalty simulation workflow and 3D aortic growth heatmaps. Linear mixed-effects regression demonstrated that TBAD growth is significantly associated with the structural stress exerted on the aortic wall. We observed a positive association between high structural wall stress regions and high growth rate regions, indicating that high structural wall stress could serve as a predictor of locations prone to rapid aortic growth. The structural stress computation workflow that incorporates patient-specific blood pressure distributions derived from CFD simulations may yield a more accurate stress field than structural mechanics-only approaches, while offering reduced computational cost compared to fully coupled FSI simulations (Rolf-Pissarczyk et al. 2025). Biomechanical simulations (Munshi et al. 2020), such as the forward penalty structural stress analysis employed in this study, can offer clinicians personalized insights into anatomical sites vulnerable to rapid aortic growth during TBAD progression. This patient-specific information adds to the current risk factor profile and allows for tailoring the treatment to the individual patient. For instance, a patient presenting with an acute uncomplicated TBAD with a proximal descending aortic diameter of 40 mm and a forward penalty analysis showing high structural wall stress in the proximal descending aorta could undergo TEVAR to reduce structural wall stress in this high-risk segment of the false lumen. This may prevent the development of a large aneurysm requiring a high-risk open procedure in the chronic phase and improve long-term survival. Conversely, structural stress data could inform the treatment of a patient with a proximal descending aortic diameter of 40 mm and low structural wall stress, who could be treated with OMT alone, and avoid the risks of stroke, spinal cord ischemia, vascular injury, and long-term adverse cardiac effects. In particular, TEVAR-induced aortic stiffening can lead to arterial hypertension, which over time may cause left ventricular hypertrophy and other complications (Azancot Rivero et al. 2024; Bakel et al. 2019; Kamenskiy et al. 2021; Vallerio et al. 2019). Due to the small sample size (9 patients) in this study, it may be challenging to establish a universal structural stress threshold for distinguishing high-risk from low-risk patients for aortic expansion and development of false lumen aneurysms. Whether such a universal structural stress threshold exists warrants further investigations. We note that early aortic growth in the subacute/early chronic phase (e.g., within 6 months) may serve as an additional predictor of long-term aortic growth rate (> 1 year) (Marway et al. 2025; Higashigaito et al. 2021). Using structural wall stress patterns and early growth outcomes as combined predictors is expected to further improve the accuracy of long-term aortic growth rate predictions.

Conventional fully coupled FSI simulations, which integrate fluid and solid mechanics solvers, provide high-fidelity biomechanical results but are often computationally prohibitive for complex geometries like patient-specific aortic dissection, requiring days to complete for a single patient (Alimohammadi et al. 2015; Chong et al. 2020). As a result, fully coupled FSI analysis is typically restricted to a very small number of patients in the literature (Munshi et al. 2020; Zimmermann et al. 2021, 2023) and may not be feasible for larger simulation cohorts due to its time-consuming nature. Moreover, these simulations often encounter convergence issues due to the high nonlinearity in geometry and material models. Heavy domain knowledge and skills are required to tackle the convergence errors. In contrast, our use of a statically determinant forward penalty model in this study enabled rapid computation of structural wall stress. The simulation run time is usually under 20 min on a desktop PC. Due to the use of static determinacy in the solid mechanics setup, this approach is robust against convergence problems. This novel, rapid forward penalty model affords the possibility of clinicians incorporating patient-specific data into their decision-making in determining the optimal therapy (OMT vs. TEVAR) for patients with acute uncomplicated TBAD.

In this study, hexahedral elements (C3D8H) were used for TBAD geometries, as they generally improve computational accuracy and efficiency compared to first-order tetrahedral elements. However, the use of hexahedral elements necessitates a more complex meshing process. As a result, image segmentation and mesh generation remain manual tasks in our current workflow and require expert human input. Although no prior experience is necessary, new users typically undergo several weeks of training to become proficient in using tools such as 3D Slicer and Hypermesh on a desktop computer. After training, image segmentation typically takes 6 h, while mesh generation requires around 12 h of expert time. To expedite the process, we are developing machine learning-based approaches to automate these tasks. Similar methods have been developed by our group and others (Pak et al. 2024, 2023; Kong and Shadden 2022) for generating patient-specific meshes in cardiovascular applications, such as whole-heart and aortic root mesh generation for transcatheter aortic valve replacement (TAVR) simulations. These approaches are expected to reduce segmentation and mesh generation time to just a few seconds. It is also worth noting that the reduced-order simulation methods employed in this study are compatible with both manual and automated workflows.

Optimal medical therapy (OMT) typically results in reduced blood pressure during the chronic phase of disease. We report the available brachial blood pressure measurements obtained within three months of the initial diagnosis for all patients in Table 4. For patients P3 ~ P9, blood pressure was significantly reduced compared to baseline due to OMT, although notable fluctuations were observed in P2, P4, P5, P7, and P9. The primary objective of this study was to assess whether baseline structural stress is spatially correlated with aortic growth rate. To ensure accurate structural stress analysis, we used the TBAD geometry from the baseline CTA and paired each scan with a temporally matched blood pressure measurement to maintain consistency between the geometric model and loading conditions. The potential influence of blood pressure fluctuations on aortic growth warrants further investigation.

**Table 4:** Available brachial blood pressure (BP) measurements of TBAD patients within three months of initial diagnosis

Patient ID		Baseline BP (mmHg)	BP follow-up time 1 (mmHg)	BP follow-up time 2 (mmHg)	BP follow-up time 3 (mmHg)	BP follow-up time 4 (mmHg)
1	SBP/DBP	159/87	152/76	158/80		
	Date	12/16/2015	12/18/2015	02/18/2016		
2	SBP/DBP	119/59	107/71	131/78		
	Date	03/18/2015	03/21/2015	06/01/2016		
3	SBP/DBP	127/61	106/53			
	Date	04/01/2008	05/13/2008			
4	SBP/DBP	147/94	143/69	91/54	123/75	139/86
	Date	04/06/2013	04/08/2013	04/10/2013	04/16/2013	05/04/2013
5	SBP/DBP	191/94	130/61	142/85	133/68	
	Date	03/25/2016	04/04/2016	04/28/2016	05/02/2016	
6	SBP/DBP	136/63	111/57			
	Date	03/15/2010	03/17/2010			
7	SBP/DBP	156/77	123/74	145/86	143/88	
	Date	04/10/2023	05/01/2023	05/16/2023	07/06/2023	
8	SBP/DBP	183/96	134/73	125/68		
	Date	01/15/2009	02/17/2009	04/13/2009		
9	SBP/DBP	168/80	128/67	140/89		
	Date	10/20/2011	10/20/2011	11/18/2011		

*SBP* systolic blood pressure, *DBP* diastolic blood pressure

### Structural wall stress vs. wall shear stress (WSS)

In this study, we computed structural wall stress, which should not be confused with WSS. WSS refers to the frictional force exerted by flowing blood on the vessel wall and is often considered a predictor of plaque formation in arterial atherosclerosis. This flow-induced frictional force is sensed by endothelial cells and transduced via mechanoreceptors into a biochemical signal that regulates vascular functionality (Peiffer et al. 2013). Changes or disturbances in WSS patterns can lead to alterations in cellular proliferation, thrombosis, and inflammation within the vessel wall. However, in the context of TBAD progression, the role of WSS requires further investigation, as the endothelium may not exist in the acutely dissected false lumen wall where aortic expansion predominantly occurs. Our findings also did not reveal a significant association between WSS and aortic growth rate, which is in line with a previous in vivo study that reported no association between WSS and aortic growth in uncomplicated TBAD (Teixido Tura et al. 2022). Furthermore, WSS typically ranges 1 ~ 10 Pa (Humphrey 2008), whereas the structural wall stress computed in this study is several orders of magnitude larger, typically around 300 kPa (Fig. 8). Structural wall stress arises from tension induced by the blood on the aortic wall, which may serve as a more relevant biomechanical regulator of aortic expansion in acute TBAD.

### Modeling assumptions and limitations

In this work, the two-way interaction between aortic wall and blood flow was not modeled. Instead, we assumed steady-state condition in the fluid domain and computed structural stress by using a reduced-order forward penalty approach. This modeling scheme was chosen due to the following considerations: (1) Although fully coupled, time-resolved FSI offers detailed hemodynamic insights, its high computational cost limits sample size (Zimmermann et al. 2023) and makes it impractical for time-sensitive clinical use. Our goal in this study is to adopt a computationally efficient approach to investigate the role of structural stress in TBAD progression in a slightly larger patient cohort. (2) We note that the dissection flap in acute TBAD can be highly compliant, exhibiting substantial motion throughout the cardiac cycle, as demonstrated in 4D flow MRI studies (Allen et al. 2019; Bäumler et al. 2020). This dynamic behavior can lead to time-dependent variations in flow patterns and pressures between the true and false lumens. Accurately capturing this behavior requires a fully coupled time-resolved FSI model with patient-specific or carefully calibrated mechanical properties of the dissection flap (e.g., Bäumler et al. 2024). However, recent studies (Deplano and Guivier-Curien 2023; Zhu et al. 2022) suggest that the pressure fields predicted by rigid-wall CFD simulations and those from two-way FSI simulations with flap motion demonstrate similar spatial patterns. A recent study (Parker et al. 2022) with a larger patient cohort has demonstrated that blood pressure fields computed from CFD simulations are predictive of TBAD clinical outcomes. Should this be the case, the structural wall stress field computed using the forward penalty approach with systolic pressure may be interpreted as a peak of the time-dependent structural stress response. Nonetheless, we acknowledge that the absence of cyclic flap motion is a limitation, and its impact warrants further investigation. (3) The mechanical properties of the dissection flap, as well as the true and false lumen walls, are highly nonlinear and patient-specific, yet largely unknown from routine CT imaging, which provides data from only a single cardiac phase. Accurately modeling nonlinear structural mechanics would require incorporating pressure-induced prestress and growth-driven residual stress. These unknown factors, along with their complex interplay, must be fully incorporated into the modeling before an accurate FSI-based hemodynamic and structural mechanics assessment could be achieved. However, comprehensively incorporating these effects from routine single-phase CT images is not feasible. (4) It has been previously demonstrated that transmurally averaged structural stress can be accurately computed within minutes using the static determinacy approach, without requiring patient-specific material properties (Liu et al. 2019b; Lu and Luo 2016; Miller and Lu 2013). Additionally, this transmurally averaged structural stress is independent of prestress and residual stress (Liu et al. 2019b). As shown in Appendix, the structural stress field obtained using the forward penalty method and that computed via a fully coupled steady-state FSI approach are nearly identical (MAPE 7.57%) for a representative TBAD geometry. However, further validation of the forward penalty method may be necessary, as TBAD anatomies can exhibit substantial inter-patient variability.

Under pulsatile blood flow, structural stress varies over the cardiac cycle, with lower stress magnitudes expected during diastole. In the present study, the fluid simulations were performed under steady-state conditions with a constant flow rate. Systolic pressure was applied to the image-derived geometry to compute peak structural wall stress, under the hypothesis that peak stress may play a critical role in driving false lumen tissue growth and remodeling (Qiao et al. 2025). We acknowledge that steady-state simulations do not capture the dynamic effects of pulsatile flow, including inertia and potential time delays between the proximal aorta and false lumen flow. While prior studies have used mean arterial pressure (MAP), calculated as MAP = (1/3 systolic + 2/3 diastolic pressure), to represent time-averaged loading conditions (Sesso et al. 2000), further research is needed to explore the use of MAP-based structural stress computations and their association with aortic growth.

The wall thickness of the TBAD geometry may be difficult to obtain from clinical CT images due to relatively limited resolutions and partial volume effect. In this study, we modeled acute TBAD and utilized the following thicknesses in the 3D TBAD model: (1) 2 mm for the true lumen wall, (2) 1 mm for the false lumen wall and dissection flap. These thickness values are consistent with our group’s experimental work with TBAD tissue specimens (Dong et al. 2022). Experimental measurements have demonstrated notable inter-patient variability in aortic wall thickness (Dong et al. 2025; Manopoulos et al. 2018) (e.g., false lumen wall thickness of 0.91 ± 0.09 mm (Dong et al. 2025)). Given that wall thickness is a critical input in structural stress computations, its variability highlights the importance of incorporating wall thickness uncertainty into the analysis. In future work, we plan to integrate experimentally derived thickness distributions into the modeling framework using uncertainty quantification methods to evaluate their impact on predicted structural stress distributions.

In this study, false lumen thrombus was modeled as a solid. Under this assumption, our results indicate that thrombus is associated with locally reduced structural stress. A similar protective effect of thrombus on aortic growth has been reported in the literature (Teixido Tura et al. 2022). However, given the small sample size, thrombus alone cannot be considered a reliable predictor of local growth rate in our analysis. Other factors, such as baseline 3D TBAD geometry, likely contribute to elevated structural stress and accelerated aortic growth. In reality, thrombus may be biphasic, consisting of both solid and fluid components. Using the minimum and maximum HU values of the aortic wall as thresholds to define the solid phase may lead to underestimation of thrombus volume. A more realistic representation should account for both phases. Furthermore, intraluminal pressure may persist within thrombus and thrombus may only partially transmit or bear pressure. This limitation warrants further investigation, ideally using modeling strategies that incorporate thrombus permeability to blood pressure (Xu et al. 2017).

Non-rigid registration between baseline and follow-up geometries extracted from CTA images presents significant challenges due to the lack of spatial landmarks and the inherently large deformations associated with aortic growth. In this study, corresponding surface nodes across time points were identified using the large deformation diffeomorphic metric mapping algorithm implemented in Deformetrica (Bône et al. 2018; Durrleman et al. 2014), which minimizes a cost function that balances geometric matching and deformation regularization. Although Deformetrica has been benchmark validated in other contexts (Goparaju et al. 2022), and Elastix has demonstrated promising performance in vascular deformation mapping (Burris et al. 2022), caution is warranted when interpreting the computed growth rates due to the absence of ground-truth spatial correspondence in CTA data.

In this work, aortic growth rate was quantified in the growth rate heatmap using %/year. Since mm/year may be more clinically relevant, we also reported the spatially averaged aortic diameter growth rate (mm/year) for each patient, calculated by averaging the radius over each circumferential layer and then across the entire descending aorta. Consequently, non-circularity cannot be assessed. In future studies, we plan to investigate additional metrics to characterize geometric features. In this work, the aorta was partitioned into 50 regions, with the descending aorta represented by regions 13–50. In future studies, we plan to use more physiologically informed partitioning and investigate growth rates in detailed anatomical regions, including the ascending thoracic aorta, aortic arch, thoracic descending aorta, and abdominal aorta (Dux-Santoy et al. 2022).

Due to the retrospective nature of this study, we acquired baseline and follow-up CT images at various intervals and calculated the temporally averaged growth rate of the outer surface of the aorta. It is important to recognize that false lumen growth is nonlinear, can vary between patients, and can differ substantially between the acute and chronic phases (Peterss et al. 2016), with slower growth rates typically observed during the chronic phase. For instance, P1 and P7 exhibited positive and negative growth rates, respectively, each with a magnitude of approximately 50% per year, whereas most other patients’ growth rates were around 5% per year. This discrepancy may be attributed to the short time interval between the baseline and follow-up CT scans for P1 (8 months) and P7 (3 months), which likely also influenced the results when follow-up time was included as a fixed-effect covariate. Although the time interval could influence the calculation of growth rates, our observation that high structural stress regions were associated with rapid growth regions remains unaffected. To better account for nonlinear growth patterns, future studies would benefit from a larger patient cohort with more uniform follow-up intervals and multiple follow-up scans per patient to enable more detailed temporal modeling. In future work, the nonlinear aortic growth rate may be analyzed separately for the true and false lumen.

We note that structural wall stress may influence aortic growth differently during the acute and chronic phases. Rapid aortic expansion is more likely to occur in the acute or subacute phase, during which the distribution of structural wall stress may have a greater impact on outcomes. In this study, we hypothesized that elevated structural wall stress during this critical period contributes more significantly to the risk of rapid aortic growth, and evaluated aortic expansion using a single follow-up CTA for each patient. Accordingly, we examined the role of structural stress distribution in the acute phase as a potential indicator of future aortic expansion. We acknowledge that structural wall stress evolves over time and may have a different effect during the chronic phase. It remains unclear whether wall stress can predict late-stage dilatation or if its influence changes across disease stages (Bäumler et al. 2024). It is also possible that structural wall stress and WSS play distinct roles at different stages of TBAD progression, which warrants further investigation.

This study serves as a pilot investigation into the role of acute structural stress in TBAD progression; therefore, a limited number of patients (*n* = 9) was analyzed. Further validation and research may be necessary before the clinical relevance of the findings can be fully established. In future work, we aim to further expand our forward penalty structural stress analysis by incorporating a larger patient cohort. With a larger dataset, we may investigate whether a universal structural stress threshold can be established to identify patients at high risk of rapid aortic expansion. Moreover, additional biomechanical markers may be identified to predict the risk of aneurysmal formation and the need for early endovascular intervention in acute uncomplicated TBAD. By further incorporating accurate constitutive description (Gasser et al. 2006; Liu et al. 2020), growth and remodeling theories (Ambrosi et al. 2019; Liu et al. 2022), in vivo material properties (Liu et al. 2017, 2018b, 2019a, c), and failure modeling (Liu et al. 2021a, b) into the modeling framework, it can be anticipated that the rate and location of aortic growth and need for intervention in acute uncomplicated TBAD may be predicted by computational simulations using information available in the initial baseline CT images.

## Conclusion

The results of this work indicated that the forward penalty workflow can be used to compute patient-specific structural wall stress distributions of acute uncomplicated TBADs in a computationally efficient manner. Our regression analysis of structural stress distributions and growth rate heatmaps revealed a significantly positive association between high structural stress regions and areas with high growth rates, which suggested that elevated structural wall stress may serve as a novel predictor of anatomic locations at risk for rapid aortic growth. Future investigations will expand upon these findings by including a larger cohort of patients to further elucidate the role of structural wall stress alongside other biomechanical markers.

## Figures and Tables

**Fig. 11:**
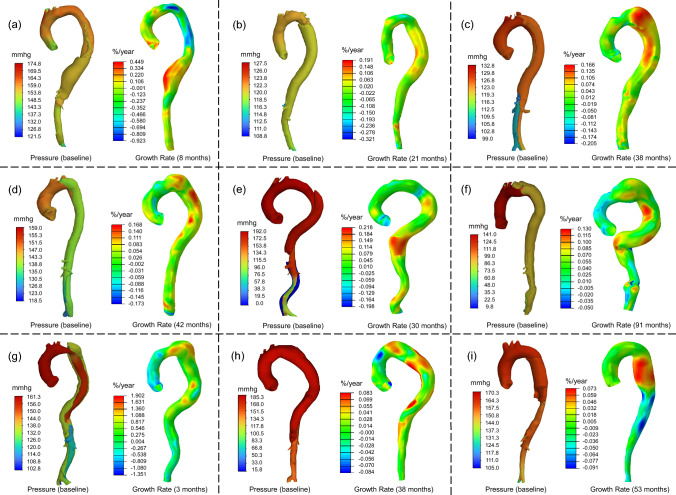
Pressure distributions and heatmaps of aortic growth rate of the TBAD patients. **a** P1; **b** P2; **c** P3; **d** P4; **e** P5; **f** P6; **g** P7; **h** P8; **i** P9

**Fig. 12:**
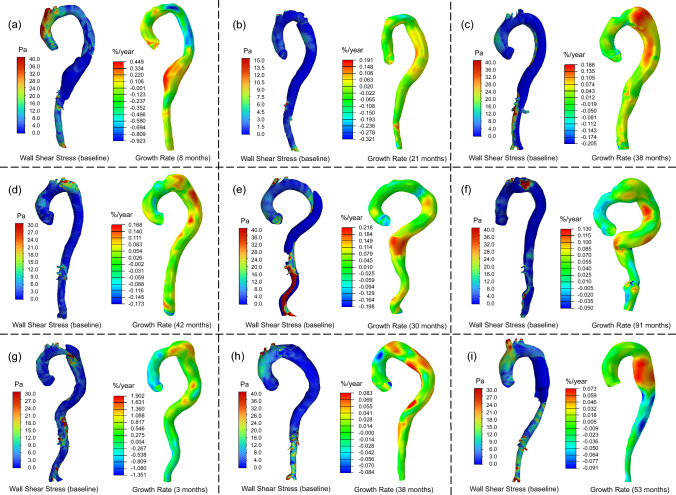
Wall shear stress (WSS) distributions and heatmaps of aortic growth rate of the TBAD patients. **a** P1; **b** P2; **c** P3; **d** P4; **e** P5; **f** P6; **g** P7; **h** P8; **i** P9

**Fig. 13:**
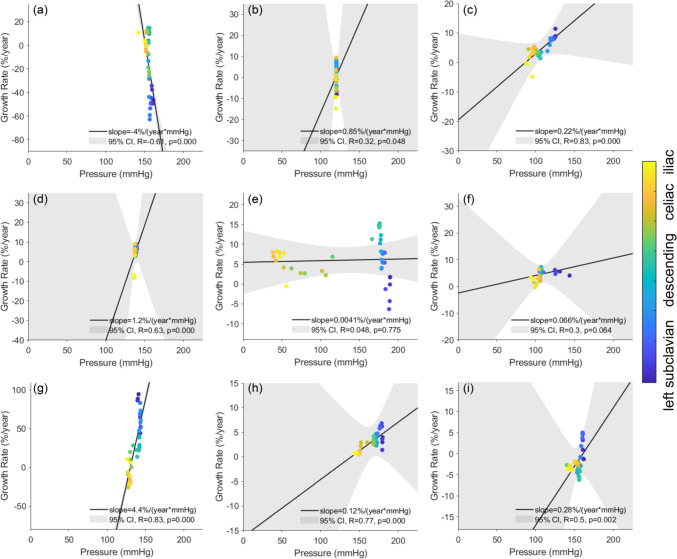
Pressure distribution and aortic growth rate of individual patients. **a** P1; **b** P2; **c** P3; **d** P4; **e** P5; **f** P6; **g** P7; **h** P8; **i** P9. Data points are color-coded by spatial location from the proximal to the distal aorta. The regression lines were obtained by using both fixed- and random-effects in the linear mixed-effects model. CI: confidence interval; R, *p*: Pearson correlation coefficient and *p* value.]

**Fig. 14:**
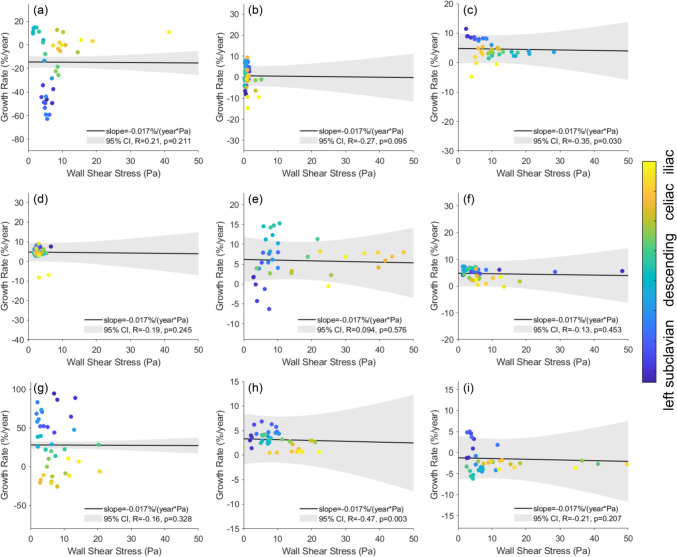
Wall shear stress (WSS) distribution and aortic growth rate of individual patients. **a** P1; **b** P2; **c** P3; **d** P4; **e** P5; **f** P6; **g** P7; **h** P8; **i** P9. Data points are color-coded by spatial location from the proximal to the distal aorta. The regression lines were obtained by using both fixed- and random-effects in the linear mixed-effects model. CI: confidence interval; R, *p*: Pearson correlation coefficient and *p* value

**Fig. 15:**
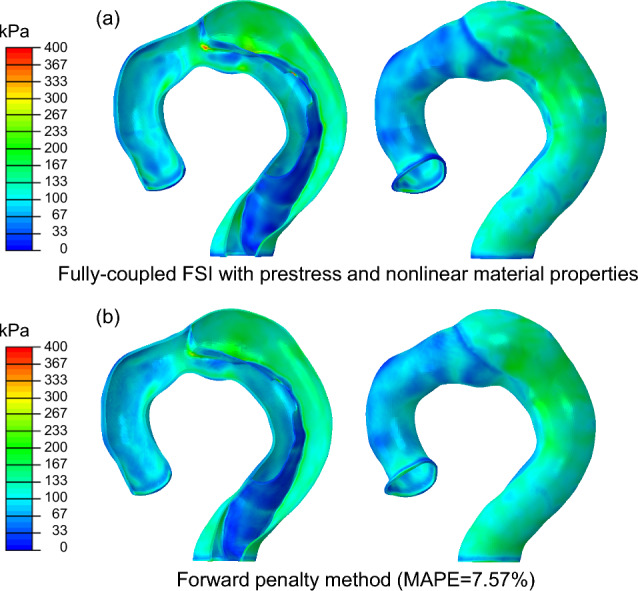
von Mises structural wall stress distribution calculated using a fully coupled steady-state fluid–structure interaction (FSI) model **a** which accounts for prestress and nonlinear material properties, compared to results from the forward penalty method **b** MAPE: mean absolute percentage error

**Fig. 16:**
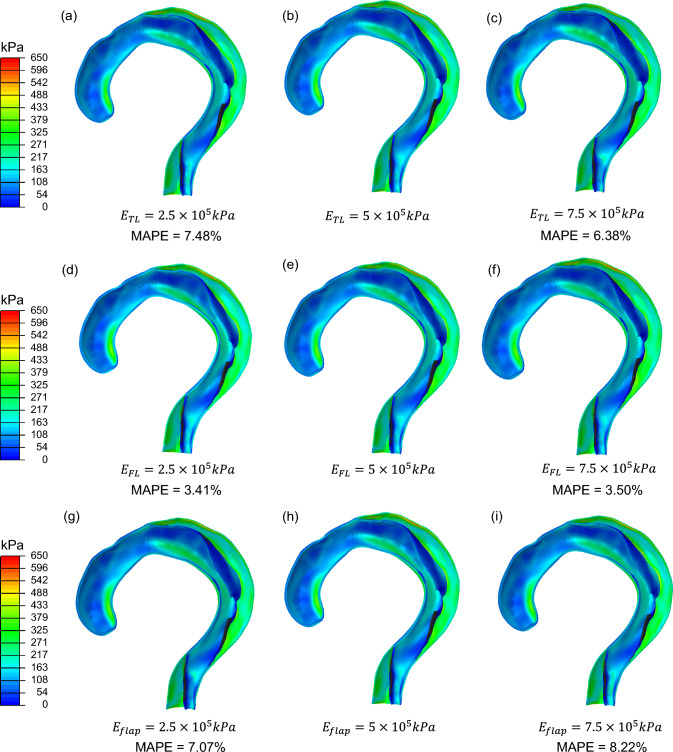
von Mises structural wall stress distributions computed by individually varying the artificial stiffness of the true lumen wall, false lumen wall, and dissection flap by  ± 50% (i.e., *E* = 2.5 × 10^5^ kPa and *E* = 7.5 × 10^5^ kPa). Panels **a**–**c** variation of true lumen stiffness; **d**–**f** variation of false lumen stiffness; **g**–**i** variation of dissection flap stiffness. MAPE: mean absolute percentage error

## Data Availability

The data that support the findings of this study are available from the corresponding author upon reasonable request.

## Appendix

### Spatial correlation analysis using pressure and wall shear stress (WSS)

Pressure distributions computed from the initial baseline CTA were compared to aortic growth rate heatmaps in Fig. 11. It is difficult to observe any clear association between pressure and growth rate. A similar comparison between WSS and aortic growth rate heatmaps is shown in Fig. 12. While WSS was elevated near regions of high velocity and narrow cross sections, such as around the primary intimal tear, WSS remained low throughout much of the false lumen, where aortic expansion typically occurs.

Figure 13 summarizes the individual correlations between pressure distributions and growth rate heatmaps for the 9 patients. Although the Pearson correlation coefficients were statistically significant in 7 out of 9 patients, the slopes varied considerably across individuals. The standard deviation of the random-effect slopes ${b}_{1m}$ was estimated at 2.14%/(year·mmHg) with a 95% CI of [1.30, 3.50]%/(year·mmHg), indicating substantial inter-patient variability. This high variability contributed to the wide CI and the non-significant *p* value for the fixed-effect association.

Figure 14 shows the individual correlations between WSS distributions and growth rate heatmaps for the 9 patients. The Pearson correlation coefficients were statistically significant in only 2 of the 9 cases. The standard deviation of the random-effect slopes ${b}_{1m}$ could not be estimated using MATLAB’s ‘fminunc’ optimization algorithm, suggesting that the variation in slopes across patients was negligible. This implies that the WSS-growth rate relationship may be adequately modeled using a common near-zero, negative slope. These findings suggest that the role of WSS in TBAD-related aortic expansion requires further investigation.

### Validation of the forward penalty structural stress computation method using a fully coupled steady-state FSI model

To validate structural stress computation using the forward penalty method, we conducted fully coupled steady-state FSI simulations that accounted for nonlinear material properties and blood pressure-induced pre-stress. In the steady-state FSI simulation, we used a representative TBAD geometry with an aortic root inlet and two outlets corresponding to the true and false lumens. The solid domain was meshed primarily with hexahedral C3D8H elements, while the fluid domain was modeled using smoothed particle hydrodynamics (SPH) with PC3D elements, allowing interaction between structural and fluid domains through contact mechanics algorithms. Based on our mesh convergence analysis, a mesh resolution of 2 mm was adopted for the solid domain. For the fluid domain, SPH particles were uniformly distributed with a spatial resolution of 0.5 mm, which is adopted from the mesh convergence results reported in Mao et al. 2016. In the FSI, the aortic tissue was modeled using the nonlinear Gasser–Ogden–Holzapfel (Gasser et al. 2006) constitutive relation, with representative material parameters derived from an experimental study (${c}_{10}, {k}_{1}, {k}_{2}, \kappa , \theta =27 kPa, 500 kPa, 0.02, 0.16, 30^\circ$) (Pham et al. 2013). As such, finite deformation was accounted for in the structural domain.

To account for pre-stress and recover the zero-pressure configuration, we employed the generalized pre-stressing algorithm (Weisbecker et al. 2014). A uniform pressure of 100 mmHg was applied to both the true and false lumen walls, and the pressure was then gradually reduced to 0 mmHg to obtain the zero-pressure geometry. The steady-state FSI simulation began from this zero-pressure configuration. A constant inlet flow rate of 25 L/min, representative of a population-averaged systolic flow, was prescribed at the aortic root. Windkessel boundary conditions were applied to both outlets, as described in Sect. 2.2. The total resistance was calculated by dividing the inlet pressure of 100 mmHg by the 25 L/min flow rate, and the outlet resistances were distributed according to Murray’s law, proportional to the cross-sectional area of each outlet. Boundary nodes at the proximal and distal ends of the aorta were fixed for the structural domain. The simulation was carried out using Abaqus/Explicit. Under the specified steady-state boundary conditions, the zero-pressure geometry was gradually inflated. Sufficient time steps were used to allow both the fluid and structural fields to reach a steady state.

For a fair comparison with the steady-state FSI results, the forward penalty approach used the steady-state, deformed geometry obtained from the FSI simulation, with the same mesh resolution and element type (C3D8H, 2 mm). An artificially stiff material (Young’s modulus = 5 × 10^5^ kPa) was assigned to the structural domain. The nonuniform pressure distribution computed from the FSI simulation was extracted and applied as a distributed load. This setup ensured consistency with the modeling procedures described in Sect. 2.2 and enabled direct comparison between the steady-state FSI and forward penalty methods.

The von Mises structural stress field, which accounts for all components of the structural stress tensor, was extracted and visualized for both the steady-state FSI and forward penalty methods. As shown in Fig. 15, the structural stress results showed only a small difference with MAPE of 7.57%, which demonstrates that the forward penalty approach achieved accurate structural stress computations in significantly less time (forward penalty: ~ 20 min vs. fully coupled FSI: ~ 40 h on a desktop computer with Intel i9-14900 K CPU). In Fig. 15, greater differences may be observed if a fully coupled, time-resolved FSI simulation is performed and compared to the forward penalty approach.

### Sensitivity analysis of artificial stiffness in the true lumen, false lumen, and dissection flap

In this study, we assumed that the true lumen, false lumen, and dissection flap share similar mechanical properties. Accordingly, a uniform, artificially stiff material property (*E* = 5 × 10^5^ kPa) was applied across the entire thoracic aortic dissection geometry. However, we acknowledge that differences in microstructural composition may exist: for example, the dissection flap is primarily composed of the intima and inner media; the wall of the false lumen includes the outer media and adventitia; and the wall of the true lumen represents the intact aortic tissue. These distinctions suggest potential variability in mechanical behavior.

To assess the impact of this variability, we conducted a sensitivity analysis, the results of which are presented in Fig. 16. Specifically, the stiffness of the true lumen wall, false lumen wall, and dissection flap was individually varied by ± 50% (i.e., *E* = 2.5 × 10^5^ kPa and *E* = 7.5 × 10^5^ kPa), based on experimental studies (Dong et al. 2025; Lou et al. 2019b) indicating that the stiffness of these regions is generally of similar magnitude. We then compared the resulting von Mises structural stress distributions to those obtained using the baseline model with uniform stiffness. The results indicated that the computed structural stress field is relatively insensitive to variations in stiffness assigned to any single component, with a MAPE below 10%.

This finding may be explained by the principle of static determinacy, which approximately holds within each individual component of the aortic wall. The equilibrium between internal forces (i.e., structural stresses) and external loads (i.e., intraluminal blood pressure) can be determined locally based on the geometry and loading conditions of each region. For example, the false lumen typically does not bear load transferred from the true lumen or dissection flap. Therefore, the resulting structural stress field depends only weakly on the specific material properties of each region.

### Partitioning of ascending and descending aorta

Using the point just distal to the left subclavian artery as a landmark, the partitioning of the ascending aorta/aortic arch and descending aorta is shown in Figure 17.

### Aortic growth heatmaps visualized on the baseline geometries

An alternative visualization of the aortic growth heatmaps, along with structural stress distributions, mapped onto the baseline geometries, is presented in Fig. 18.
